# Self‐directed self‐management interventions to prevent or address distress in young people with long‐term physical conditions: A rapid review

**DOI:** 10.1111/hex.13845

**Published:** 2023-08-21

**Authors:** Nadia Corp, Lucy Bray, Carolyn A. Chew‐Graham, Kay Polidano, Tamsin Fisher, Adam D. Farmer, Megan McDermott‐Hughes, Benjamin Saunders

**Affiliations:** ^1^ School of Medicine Keele University Staffordshire UK; ^2^ School of Nursing, Midwifery and Allied Health, Faculty of Health, Social Care and Medicine Edge Hill University Ormskirk UK; ^3^ Department of Sociology University of Malta Msida Malta; ^4^ Department of Gastroenterology University Hospitals of North Midlands NHS Trust Stoke‐on Trent UK

**Keywords:** adolescents, chronic disease, distress, mental health, rapid review, self‐management, young adults

## Abstract

**Background:**

Comorbid distress in adolescents and young adults with physical long‐term conditions (LTCs) is common but can be difficult to identify and manage. Self‐directed self‐management interventions to reduce distress and improve wellbeing may be beneficial. It is unknown, however, which intervention characteristics are successful in supporting young people. This rapid review aimed to identify characteristics of self‐directed self‐management interventions that aimed, in whole or part, to address distress, wellbeing or self‐efficacy in this population.

**Methods:**

A systematic search was conducted for relevant controlled studies in six databases. Data on study settings, population, intervention characteristics, outcome measures, process measures and summary effects were extracted. The risk of bias was assessed using the Cochrane Risk of Bias tool v1, and the strength of evidence was rated (informed by Grading of Recommendations, Assessment, Development and Evaluations). Patient and public involvement members supported the review process, including interpretation of results. The rapid review was registered with PROSPERO (ID: CRD42021285867).

**Results:**

Fourteen studies were included, all of which were randomised trials. Heterogeneity was identified in the health conditions targeted; type of intervention; outcome measures; duration of intervention and follow‐up. Three had distress, wellbeing or self‐efficacy as their primary outcome. Four modes of delivery were identified across interventions—websites, smartphone applications, text messages and workbooks; and within these, 38 individual components. Six interventions had a significant benefit in mental health, wellbeing or self‐efficacy; however, intervention characteristics were similar for beneficial and non‐beneficial interventions.

**Conclusions:**

There is a paucity of interventions directly targeting distress and wellbeing in young people with physical LTCs. In those identified, the heterogeneity of interventions and study design makes it difficult to identify which characteristics result in positive outcomes. We propose the need for high‐quality, evidence‐based self‐management interventions for this population; including (1) more detailed reporting of intervention design, content and delivery; (2) robust process evaluation; (3) a core outcome set for measuring mental health and wellbeing for self‐management interventions and (4) consistency in follow up periods.

**Public Contribution:**

Seven young people with an LTC were involved throughout the rapid review, from the development of the review protocol where they informed the focus and aims, with a central role in the interpretation of findings.

## 1 BACKGROUND

Individuals with long‐term physical health conditions (LTCs) are at increased risk of experiencing distress and low mood, which, if not appropriately managed, can lead to depression.^1^ Distress can be defined as symptoms ‘severe enough to warrant consultation’, but where clinicians have not diagnosed depression or other mental health conditions.^2^ Comorbid distress in individuals with physical LTCs may be particularly problematic in later adolescence and young adulthood, for example, between the ages of 16 and 29, as individuals are also undergoing major life transitions, such as moving away from home, going to college/university or beginning fulltime employment, which can make managing an LTC even more challenging.^3^, ^4^ Furthermore, comorbid mental health problems can be difficult to identify and manage among this population in healthcare settings, where the focus may be on the management of physical symptoms.^5^, ^6^ Interventions targeted at this age group that aim to prevent or reduce comorbid distress are therefore important. Given the increased pressure on healthcare services globally, self‐management interventions focused on supporting young people to manage and reduce distress may be particularly beneficial.^7^

Self‐management can be defined as ‘the systematic process of learning and practicing skills which enable individuals to manage their health condition on a day‐to‐day basis, through practicing and adopting specific behaviours which are central to managing their condition, making informed decisions about care, and engaging in healthy behaviours to reduce the physical and emotional impact of their illness’.^8^ Encouraging self‐management for people with LTCs is part of the UK National Health Service Long Term Plan, with the aim to ‘support and empower people to manage their ongoing physical and mental health conditions themselves’.^9^ ^(^^p.93)^ In keeping with this agenda, self‐directed self‐management interventions, through which young people develop knowledge and skills to manage their health without the need for ongoing direction from healthcare professionals (HCPs), could help to reduce distress in young people with physical LTCs while also helping to ease the burden on healthcare services.^7^, ^10^

It is unknown, however, which characteristics of self‐management interventions for young people with physical LTCs are successful in reducing distress, as well as improving wellbeing. This is the case both with interventions that directly target these outcomes and those which may have a secondary impact on distress and wellbeing. Wellbeing is defined by the UK Department of Health and Social Care as ‘feeling good and functioning well and comprises an individual's experience of their life; and a comparison of life circumstances with social norms and values’.^11^ ^(^^p.6)^ Gaining a better understanding of the characteristics and modes of delivery of interventions in this population, particularly those with positive outcomes, is important for informing the development of future research and practice development.

This rapid review aimed to identify characteristics associated with self‐directed self‐management interventions that aimed, in whole or part, to address distress, wellbeing or self‐efficacy in young people with physical LTCs. Self‐efficacy was included, as greater self‐efficacy has been found to be important for positive mental health and wellbeing in younger people with LTCs.^12^ We aimed to identify any differences in mode of delivery and intervention components between interventions that did and did not demonstrate positive effects for these outcomes.

The review is part of a larger study—the UK National Institute for Health Research‐funded Stoma Support Study, which aims to co‐design with young people and HCPs, an intervention to provide support for managing distress in young people with inflammatory bowel disease (IBD) who have undergone stoma surgery. This is where all or part of, the large bowel, is removed resulting in an opening in the abdomen through which faeces are collected in a bag attached to the skin. One part of this intervention will be a self‐directed self‐management intervention to be used by young people. This builds on earlier work which identified that distress experienced by young people after stoma surgery was often not identified or effectively addressed in healthcare settings, and young people identified a need for age‐appropriate online resources to support self‐management of stoma‐related distress.^6^, ^13^ This rapid review can therefore inform the future design of self‐management interventions for young people with physical LTCs in general, and more specifically, will help to inform the design of a future Stoma Support intervention.

### 1.1 Patient and public involvement (PPIE)

Central to this rapid review was the role of PPIE. Our PPIE group, which we established purposefully for the associated study linked to this review, was involved at various stages of this rapid review—from the development of the review protocol where PPIE members informed the focus and aims of the review to the interpretation of the review findings. The details of this group and their contributions will be outlined later in Section 2.6. The benefits of incorporating PPIE into systematic reviews have been highlighted widely in previous literature.^14^, ^15^, ^16^ Harris et al. propose that PPIE involvement in reviews leads to a broadening of what we might consider to be ‘expertise’, incorporating the experiential expertise of patients into reviews that have been ‘traditionally undertaken by ‘certified’ experts (by virtue of educational qualification)’.^17^ ^(^^p.211)^ In this review, we aimed to draw strongly on this experiential expertise to both support the interpretation of the review findings, as well as to draw out their significance and implications.

## 2 METHODS

A rapid review was conducted and reported, informed by Cochrane guidance for rapid reviews,^18^ the Preferred Reporting Items for Systematic Reviews and Meta‐Analysis guidance 2020^19^ and Synthesis Without Meta‐analysis in systematic reviews reporting guidelines.^20^ An a priori protocol was written and registered with the International prospective register of systematic reviews (PROSPERO ID: CRD42021285867).

### 2.1 Search strategy

A comprehensive search strategy was designed and conducted by an information specialist (N. C.) to identify eligible studies. Six electronic bibliographic databases were searched: MEDLINE (OVID), EMBASE (OVID), Cochrane CENTRAL, HMIC (OVID), CINAHLPlus (EBSCO) and PsycINFO (EBSCO) from database inception to 18 October 2021. The search strategy utilised both subject headings and free text searching, combining terms for self‐management, long‐term conditions (LTCs), and controlled studies (see Supporting Information: File 1 for all database searches). In addition, the reference lists of relevant systematic reviews identified during screening were checked to identify eligible studies.

The results of each search were downloaded into Endnote™ 20 (reference management software; Clarivate Analytics, available at www.endnote.com) to facilitate deduplication, with the resulting unique records being imported into Covidence systematic review software (Veritas Health Innovation, available at www.covidence.org) for screening.

### 2.2 Eligibility criteria

To be eligible for inclusion, studies needed to be of a controlled design, that is (non‐)randomised controlled trials, or other designs with a control group or period (i.e., cohort studies, before‐and‐after studies or interrupted time series). Studies reporting self‐management interventions that were self‐directed were included, that is, did not involve HCPs or trained peer input as a routine part of the intervention received by all participants. Group interventions were also excluded. To capture as many self‐directed interventions as possible, inclusion criteria were expanded to include studies comparing self‐directed self‐management to any control, rather than just usual care, no (additional) intervention or with attention control, as stipulated in the protocol. Studies were included if they reported on any self‐directed self‐management intervention developed for young people (16–29 years old) with long‐term physical conditions, which aimed, in whole or part, to prevent or address distress/mental health needs. A scoping search revealed a paucity of studies targeting this age group, and therefore it was decided to include studies where the study population's mean age ±1 standard deviation fell within the age range of interest, or if a subgroup analysis by age was conducted so data on young people could be extracted.

Studies were included if they reported at least one of the primary outcomes of interest (psychological distress including measures of anxiety and depression, well‐being including quality of life, and life satisfaction measures, confidence, self‐efficacy and knowledge regarding management of distress/mental health/wellbeing). Studies reporting only secondary outcomes of interest (i.e., acceptability, preference and satisfaction), and no primary outcomes were excluded.

Given the time restraints on a rapid review, only English‐language publications were included.

### 2.3 Study selection

To expedite the review process, N. C. screened all titles and abstracts against eligibility criteria, with the first 150 records independently screened by a second reviewer (B. S.) to check accuracy. The agreement was 90% and after discussion, further refinement of the eligibility criteria was made. All excluded titles and abstracts were also scanned by a second reviewer (B. S. or L. B.) to ensure no studies had been excluded in error. Similarly, full texts were screened by a single reviewer, with all included and excluded full texts checked by a second reviewer. Any disagreements were resolved through discussion. Reasons for exclusion at the full‐text stage were recorded.

### 2.4 Data extraction and risk of bias

A customised data extraction form was developed in Excel, piloted and used to record all data required for analysis. Data from included studies were extracted on: country and healthcare setting; population characteristics; characteristics of the intervention, informed by the TIDieR checklist,^21^ such as theories underpinning the intervention, delivery mode and tailoring; outcome measures; process measures (intervention adherence and fidelity) and summary effect estimates were reported. Concurrent with data extraction, the risk of bias was appraised using the Cochrane Risk of Bias tool v1.^22^

Data extraction was undertaken by one reviewer and checked by a second for correctness and consistency. Discrepancies were resolved through discussion.

### 2.5 Data analysis and evidence synthesis

The aim of the evidence synthesis was not to estimate the overall effectiveness of self‐management interventions and consequently, a meta‐analysis was not conducted. Evidence was synthesised narratively and focused on identifying characteristics associated with self‐directed self‐management interventions that were demonstrated to have positive effects primarily on the review's outcomes of interest, such as mode of delivery and intervention components. The synthesis began with a description of included study characteristics and main results presented in summary tables. Heterogeneity, that is, variability among studies, was informally assessed with consideration of study designs, intervention characteristics (most notably mode of delivery) and LTC of the study population.

The components making up each intervention were extracted and tabulated to facilitate comparison between different types of intervention and effective versus noneffective interventions. The narrative synthesis explored similarities and differences in the study results in relation to the characteristics of the studies, with particular reference to intervention characteristics, that is, mode of delivery and intervention components, and their methodological quality.

To rate the overall certainty of evidence associated with each intervention characteristic identified, a set of criteria was devised, inspired by the Grading of Recommendations, Assessment, Development and Evaluations methodology.^23^ This approach considered five factors that influence confidence in the body of evidence, and criteria were devised to identify concerns related to each (Box 1). The overall certainty of evidence for each intervention characteristic was then rated (Box 2).

Box 1 **Concerns relating to five factors influencing confidence**

1. Number of studies—if less than 3;
2. risk of bias—if at least half of RCTs for a characteristic have high or unclear risk associated with allocation concealment, incomplete outcome data or selective reporting;
3. consistency—if under half of the studies show statistically significant benefit;
4. precision—if at least half of the studies have a sample size of <50 per intervention arm;
5. and applicability—if mean age falls out with the age range of interest (but within one standard deviation) and no outcome of interest to the review is reported as the primary outcome in the study.

Box 2 **Rating of overall certainty of evidence**

1. High, if there were no concerns with any factor, that is, multiple trials with no study limitations, findings are consistent (i.e., at least half of studies show statistically significant benefit), sample size at least 50 per arm and directly applicable to review question;
2. moderate, if there was concern with any one factor;
3. low, if concerns with two or three factors; and
4. and very low, if concerns regarding any four or five factors.

### 2.6 PPIE group input in interpreting results

**Figure 1:**
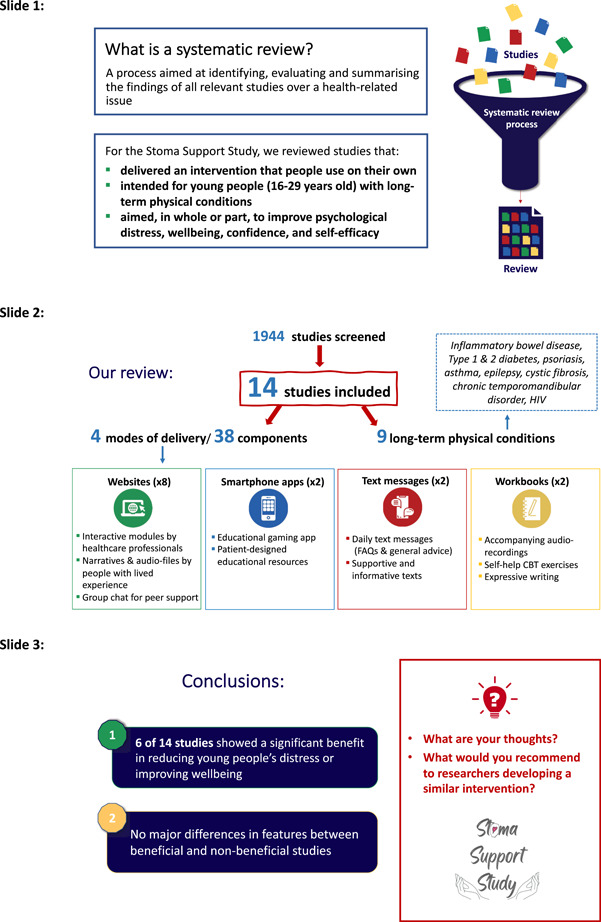
Visual summary of the review process and results for patient and public involvement. Abbreviations: FAQs, frequently asked question.

Following the completion of the evidence synthesis, a visual summary of the review process and results were sent to members of the associated study's PPIE group (see Figure 1). A 1‐h meeting was then held, via video platform, with seven PPIE members, all of whom had undergone stoma surgery due to IBD. Five members identified as female, and two as male, aged between 19 and 37 years, and lived in a range of regions in England. Initially, two members of the research team, B. S. and K. P., talked through this visual summary and then presented a lay summary of each of the studies included in the review. The research team's analysis was not presented, to ensure that group members did not feel the need to shape their interpretation of the review results according to the research teams'. Beyond just contributing feedback, the group's input was considered integral to drawing out implications and forming conclusions. The PPIE group helped interpret the findings of the review, including the context around why individual interventions either were or were not successful in improving young people's outcomes. The group also had a key role in helping to identify lessons from the findings for future self‐directed self‐management interventions for young people with LTCs, that is, their answer to the ‘so what?’ question. More specifically, they helped us to identify the implications of findings for the development of a future self‐management intervention to support young people with an IBD stoma. The interpretation of the results by the PPIE group is outlined later in the Discussion (Section 4.2).

## 3 RESULTS

### 3.1 Study flow

**Figure 2:**
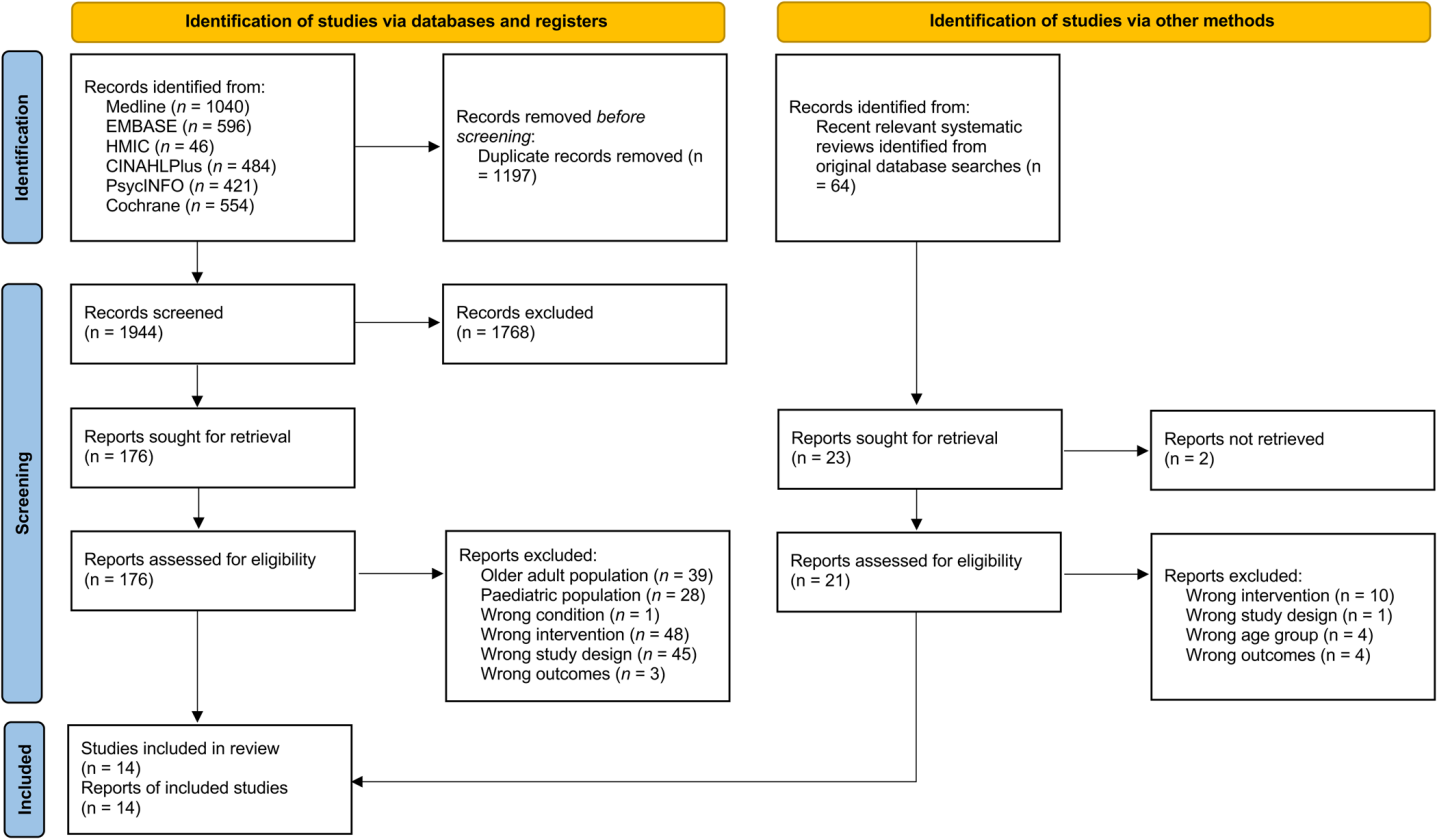
Preferred Reporting Items for Systematic Reviews and Meta‐Analyses 2020 flow diagram. From: Page et al.^19^ For more information, visit: http://www.prisma-statement.org/.

The search identified 1944 unique records, of which 176 were included for full‐text screening. Of these, 12 met the inclusion criteria and 162 were excluded for the reasons given in Figure 2. A further 21 full texts were assessed for eligibility after being identified from relevant systematic reviews picked up by the original database search. This led to the inclusion of an additional two relevant studies (Figure 2). In total, 14 studies were included in this rapid review.^24^, ^25^, ^26^, ^27^, ^28^, ^29^, ^30^, ^31^, ^32^, ^33^, ^34^, ^35^, ^36^, ^37^

### 3.2 Characteristics of included studies

The study characteristics of the included studies are detailed in Table 1. There was a high level of heterogeneity in terms of the LTCs under study; type of self‐directed self‐management intervention; outcome measures and duration of intervention and length of follow‐up. All included studies were either RCTs (*n* = 11) or pilot RCTs (*n* = 3). No studies using other controlled study designs were identified. Eleven studies (79%) used waitlist, standard care or attention controls, with the remaining three studies having active controls. Most studies were conducted in the United States (*n* = 7, 50%), with slightly fewer in Europe (*n* = 6, 43%) and one in Australia (7%), and were published between 2007 and 2021. The mean sample size was 128 individuals (range 40–329), with almost two‐thirds of studies (*n* = 9, 64%) reporting a greater percentage of females (range 21.3%–100%). LTCs were varied: Four studies (29%) concerned type I diabetes mellitus (T1DM, *n* = 4, 29%), two studies (14%) each focused on IBD and asthma and single studies (7% each) addressed type 2 diabetes, HIV, epilepsy, chronic temporomandibular disorder, or mixed chronic diseases (i.e.,  IBD, cystic fibrosis, T1DM). Studies ranged in the length of follow‐up from 1 day to c. 15 months, with the majority running for between 3 and 6 months (*n* = 9, 64%). Regarding the outcomes of interest to this review, the primary outcomes of psychological distress, well‐being and self‐efficacy outcomes were each reported in half of the studies (*n* = 7), while secondary outcomes were reported in three studies (satisfaction *n* = 2, acceptability *n* = 1, preference *n* = 0).

**Table 1:** Study characteristics of included trials.

Reference, country, study design	Condition	*n*	Age years mean (SD)	% Female	Interventions	Length of follow‐up	Outcome (type): Outcomes of interest reported	Comments on significant treatment effects and secondary outcomes, other study outcomes
Ayar et al.^24^ Turkey RCT	Type I diabetes	70	14.3 (1.76)	NR	SM: ‘Youth diabetes’—web‐based diabetes education + standard care Control: Standard care	6 months	*Well‐being*: Pediatric QL Inventory ^™^ 3.0 Diabetes Module *Self‐efficacy*: Diabetes management self‐efficacy scale in adolescents with type 1 diabetes mellitus	The significant treatment effect for both outcomes at 3 and 6 months, in favour of ‘Youth diabetes’ *Other outcomes*: A1C
Balato et al.^25^ Italy RCT (pilot)	Psoriasis	40	38.9 (9.86)	45%	SM: Text messages Control: No (additional) intervention	12 weeks	*Well‐being*: Dermatology Life Quality Index *Satisfaction*: Usability and satisfaction of the text messaging system	Significant treatment effect in favour of text message intervention. 85% found text messages useful, 75% would continue to use and recommend to a friend *Other outcomes*: Skin severity (PASI, BSA, PGA); patient‐perceived disease severity (SAPASI); Patient‐physician relationship; treatment adherence
Bell et al.^26^ United States RCT	Type I diabetes	191	14.6 (1.55)	51%	SM: Narrative care messages Control: Standard care message	1 day	*Psychological distress*: Positive emotion; stress and burnout perceptions *Self‐efficacy*: Self‐Efficacy for Diabetes Management Scale; behavioural intention (interpersonal‐ and action‐based behaviours)	*Other outcomes*: Perceived message effectiveness; outcome expectation (positive, negative)
Chapman et al.^27^ United Kingdom RCT (pilot)	Inflammatory bowel disease	329	36.8 (9.14)^a^ Median: 36.3 Range: 18.5–73.0	72.3%	SM: Tailored digital intervention to change adherence‐related beliefs and barriers Control: Standard care	3 months	*Psychological distress*: HADS‐Anxiety; HADS‐Depression *Well‐being*: SIBDQ *Acceptability*: Acceptability of digital intervention	Significant treatment effect for outcomes at 3 months, in favour of tailored digital intervention. Overall, the digital intervention was acceptable to participants: feedback was mainly positive regarding intervention content, website function, and perceptions of the intervention source. *Other outcomes*: BMQ b; VASA; MARS; PSM; SIMS AU; SIMS PP; IPQ
Dilorio et al.^28^ United States RCT	Epilepsy	194	40.9 (13.32)	73.6%	SM: ‘WebEase’—online epilepsy self‐management programme aimed at medication adherence, managing stress, and improving sleep Control: Waitlist	12 weeks	*Psychological distress*: Perceived Stress Scale^b^; Revised Epilepsy Stressor Inventory^b^ *Self‐efficacy*: Epilepsy Self‐Efficacy Scale *Well‐being*: Quality of Life in Epilepsy Scale‐10	*Other outcomes*: medication adherence^b^; sleep quality^b^; self‐management, knowledge. Nb: Those completing ≥1 WebEase module showed significantly greater self‐efficacy, with a trend toward significance observed for medication adherence, perceived stress, self‐management and knowledge
Hockemeyer and Smyth^29^ United States RCT	Asthma	54	20.7 (5.37)	53.7%	SM: Self‐administered manual‐based stress management intervention for asthma Control: ‘Placebo’ manual	4 weeks	*Psychological distress*: Perceived Stress Scale	*Other outcomes*: Lung function (FEV1) b; Workbook credibility
Huang et al.^30^ United States RCT	Inflammatory bowel disease; Cystic fibrosis; Type 1 diabetes	81	16.9 (1.65)^a^ Median: 17 Range: 12‐20	54%	SM: MD2Me—technology‐based disease management intervention Control (active): Monthly mail/email messages re general health. Disease‐specific info is given as needed. Usual modes of communication are open	8 months	*Well‐being*: QOL *Self‐efficacy*: Patient Activation Measure (PAM)	MD2Me recipients showed significant improvement in PAM across the study period compared to controls. *Other outcomes*: Chronic disease management (TRAQ), patient initiated communication
Hunt et al.^31^ United States RCT	Inflammatory bowel disease	140	35.6 (13.18)	66%	SM: Self‐help CBT book Control (active): Active psychoeducation workbook (designed to cover some material relevant to depression and anxiety but did not include any specific CBT interventions)	3 months	*Psychological distress*: Gastrointestinal Cognitions Questionnaire b; Spielberger State‑Trait Anxiety Inventory b; Beck's Depression Inventory II^b^; Visceral Sensitivity Index^b^ *Well‐being*: Short Inflammatory Bowel Disease Questionnaire b	Treatment effect at 6 weeks in favour of Self‐help CBT book. No control at 3 months but maintained benefit from baseline. *Other outcomes*: disease activity and severity (HBI; GSRS)^b^
Joseph et al.^32^ United States RCT	Asthma	314	15.3 (1.0)	63.4%	SM: ‘Puff City’—multimedia web‐based asthma management programme Control: Existing generic asthma websites	12 months	*Well‐being*: Quality of life	*Other outcomes*: Symptom days in last 2 weeks b; symptom nights; days of restricted activity; days of changed plans; school days missed in last 30 days; asthma‐related emergency department visits and hospitalisations in last 3 months; Core behaviours
Klee et al.^33^ Switzerland RCT	Type I diabetes	55	13.6 (2.4)	43.6%	SM: ‘Webdia’—patient‐designed DIY mHealth app Control: Usual care	3 months	*Well‐being*: Diabetes QoL for Youth questionnaire *Satisfaction*: satisfaction with app	Satisfaction was high: 39.3% rated the programme as ‘excellent’ and 46.4% as ‘good’. *Other outcomes*: HbA1c b (in favour of Webdia); hypoglycaemic events
Lam et al.^34^ Sweden RCT (pilot)	Chronic temporo‐mandibular pain disorder	43	29.1 (10.74)^a^ Median: 27 IQR: 23‐37	79%	SM: Internet‐based multimodal pain programme Control (active): Occlusional splint (Hard Michigan‐type stabilisation splint placed on upper jaw)	6 months	*Psychological distress*: Patient Health Questionnaire‐9; Generalized Anxiety Disorders‐7; Perceived Stress Scale‐10; Pain Catastrophising Scale	Control is active but not SM. Concluded high attrition rate demonstrated a RCT with this design was not feasible. *Other outcomes*: Graded Chronic Pain scale (for pain intensity, pain‐related disability),^b^ jaw functional limitation scale‐8^b^
Linden et al.^35^ Sweden RCT	Type I diabetes during pregnancy and early motherhood	174	30.7 (4.51)	100%	SM: Person‐centred web‐based support + standard care Control: Standard care	Early pregnancy to 6 months post childbirth c. 15 months	*Mental/psychological distress*: Swedish version of the Problem Areas in Diabetes Scale (SWE‐PAID‐20) *Self‐efficacy*: Swedish Diabetes Empowerment Scale, short version (SWE‐DES‐10)^b^ *Well‐being*: 12‐item well‐being questionnaire (W‐BQ12)^b^	*Other outcomes*: Self‐perceived health; SOC‐13; Swedish Hypoglycaemia Fear Survey. Evaluation of web‐based support (structured questionnaire with free‐text alternative)
Middleton et al.^36^ Australia RCT	Type 2 diabetes	40	32.7 (5.18)	50%	SM: Enhanced SMS text‐based support and reminder programme + standard care Control: Standard care	12 months	*Psychological distress*: Problem Areas in Diabetes 5‐item Short Form (PAID‐5) *Self‐efficacy*: Diabetes Empowerment Scale‐Short Form c	Significantly greater proportion of participants recording favourable change in DES‐SF at 6 months (relative to baseline) in SM arm. No difference detected at 12 months. Nb: no difference when considering actual scores. *Other outcomes*: Clinical attendence^b^; metabolic indices (HbA1c, BMI, lipids); Diabetes self‐management practice; Pathology results available at clinic visits; Type 2 Diabetes Stigma Assessment Scale (DSAS‐2)
Whiteley et al.^37^ United States RCT	HIV	66	22.4 (2.50)	21.3%	SM: ‘BattleViro’—iPhone gaming adherence intervention with game related text messages Control: Non‐HIV‐related game	16 weeks	*Self‐efficacy*: Self‐efficacy for ART use	*Other outcomes*: HIV‐1 viral load; ART adherence (self‐report and by electronic pill dispensing device); HIV and ART knowledge scales; ART motivation; social support

*Note*: Underlined intervention—indicates a significant intervention effect on outcomes of interest compared to control. Underlined outcome—indicates outcome where significant treatment effect detected (*p* < .05).

Abbreviations: ART, antiretroviral treatment; BMI, body mass index; BMQ, Beliefs about Medicine Questionnaire; BSA, body surface area; FEV1, forced expiratory volume in the first second; GSRS, Gastrointestinal Symptom Rating Scale; HADS, Hospital Anxiety and Depression Scale; HBI, Harvey–Bradshaw Index; IPQ, Brief Illness Perception Questionnaire; IQR, interquartile range; LSSS‐3, Liverpool Seizure Severity Scale; MARS, Medication Adherence Report Scale; *n*, sample size at randomisation; NR, not reported; PAM, Patient Activation Measure; PASI, Psoriasis Area and Severity Index; PGA, Physicians Global Assessment; PSM, Perceived Sensitivity to Effects of Medicines; QoL, Quality of life; RCT, randomised controlled trial; SAPASI, Self‐Administered Psoriasis Area Severity Index; SD, standard deviation; SIBDQ, Short Inflammatory Bowel Disease Questionnaire; SIMS AU, Satisfaction with Information about Medicines Action and Usage Subscale; SIMS PP, Satisfaction with Information about Medicines Potential Problems Subscale; SM, self‐management; SOC‐13, 13‐item Sense of Coherence Questionnaire; TRAQ, Transition Readiness Assessment Questionnaire; VASA, Adherence Visual Analogue Scale.

^a^ Mean and standard deviation estimated from median and range or IQR after Luo et al 2018 and Wan et al 2014, respectively, using https://www.math.hkbu.edu.hk/~tongt/papers/median2mean.html.

^b^ Primary study outcomes.

^c^ Significance regarding proportion of individuals recording favourable change in score from baseline, not actual/change in score.

Of the 14 interventions identified, only 6 were found to have a significant treatment benefit compared to control with respect to this review's primary outcomes of interest,^24^, ^25^, ^27^, ^30^, ^31^, ^36^ however, a further 3 interventions were shown to have a significant treatment benefit on their specified primary outcomes^29^, ^32^, ^33^.

### 3.3 Intervention characteristics

The primary mode of delivery for self‐management interventions was web‐based (*n* = 8), with the remaining interventions' main mode of delivery equally distributed between mobile device apps (*n* = 2), text messages (*n* = 2) and workbooks (*n* = 2). However, most interventions utilised multiple modes of delivery (*n* = 9) which included one or more of the following additional modes: web‐based group/peer discussion; phone calls; emails; text messages; audiovisual recordings; an initial face‐to‐face meeting and a complementary workbook (see Tables 2 and 3; full details in Supporting Information: File 2). Three interventions incorporated automated elements such as algorithms to manage mobile text messaging,^36^ to determine web‐based personalised messages,^27^ or to provide disease management support and access to the healthcare team if necessary.^30^

**Table 2:** Characteristics of interventions.

Reference	Intervention	Study aim, theories/frameworks informing the intervention	Mode of delivery	Intervention providers	Location(s) where intervention occurred	Intervention intensity and duration	Tailoring	Fidelity of delivery
Ayar et al.^24^	‘Youth diabetes’: web‐based diabetes education + standard medical care	To examine whether web‐based diabetes education was effective in improving metabolic control, self‐efficacy for diabetes self‐management, and QoL in adolescents with T1DM.	Online; individual, plus group area enabling patients to chat—accessible at any time	Researchers determined and updated website information and suggested topics for blog discussions (administrative)	NR	Delivered over 6 months. Participants encouraged to log in ≥2 times a week to update blogs. A reminder message was sent each time new material was added to the website.	No	NR
Balato et al.^25^	Text messages	To evaluate the use of text messages in improving treatment adherence and patient outcomes, e.g., QoL, disease severity, patient‐perceived disease severity and the patient–physician relationship.	Text messages; individual	Investigators responsible for sending text messages (administrative)	NR	12 weeks. 1 text message daily in the same randomly selected order (reminders 3× week, education tools 4× week).	No	NR
Bell et al.^26^	Narrative care messages	To determine if narratives would provide a better tool to improve disease management for adolescents with T1DM.	Online; individual	N/A	NR	One off, immediate response.	No	NR
Chapman et al.^27^	Tailored digital intervention to change adherence‐related beliefs and barriers	The study aimed to develop and assess a tailored digital intervention (algorithm) to support adherence to maintenance treatment for patients with IBD. The intervention employed a Perceptions and Practicalities Approach and was informed by Horne's Necessity‐Concerns Framework and utilised several Behaviour Change Techniques.	Online; individual including online video (optional)	Automated (algorithm)	NR	Over 3 months, at patients' convenience.	Yes	Yes
Dilorio et al.^28^	WebEase: theory‐based, interactive, internet‐based self‐management programme for people with epilepsy	The study aimed to determine if individuals with epilepsy who participated in WebEase programme demonstrated improvements in medication adherence, perceived stress, and sleep quality. WebEase created to provide self‐management education and support for people with epilepsy. It was based on self‐management models and self‐determination principles and incorporated concepts and principles from three theoretical perspectives: social cognitive theory, the transtheoretical model of behaviour change, and motivational interviewing.	Online; individual including online audio‐files (optional)	N/A	NR	6 weeks. Programme set so each participant spent 2 weeks in each of the 3 core modules (medication, stress, sleep management).	No	NR
Hockemeyer and Smyth^29^	Self‐administered manual‐based stress management intervention	To develop and examine the feasibility and effectiveness of a complementary, self‐administered, manual‐based intervention for asthmatic college students, which incorporated three major treatment components: relaxation training, CBT, and written emotional expression.	Workbook; individual, plus accompanying audiocassette	N/A	At home or residence	4‐weeks. CBT/problem‐solving skills completed on the first or second day of the week in each of the 4 weeks. 1–2 days later, instructed to complete the writing exercises. In own time.	No	Yes
Huang et al.^30^	MD2Me— technology‐based disease management intervention	The study aim was to evaluate a generic, internet‐ and mobile phone‐delivered disease management intervention aimed at improving disease management, self‐efficacy, and communication in adolescents with chronic disease (IBD, CF, T1DM—chosen to represent broad disease spectrum). Intervention based on Bandura's Social Cognition Theory, targeting self‐management constructs of disease symptom monitoring, responding to monitoring with appropriate treatments, and actively working with healthcare providers to manage care.	Online and text messages; individual	Automated SMS algorithm provided disease management support and access to healthcare team	NR	First 2 months: log on to website weekly; and received tailored SMS 3–5 a week.	Yes	Yes
Hunt et al.^31^	Self‐help CBT book	To determine the effectiveness of a self‐help CBT workbook for patients with IBD.	Self‐help book; individual	N/A	NR	As and when over 6 weeks.	No	NR
Joseph et al.^32^	Puff city— multimedia, web‐based asthma management programme	The study aimed to develop and evaluate a multimedia, web‐based asthma management programme (Puff City) to specifically target urban high school students. To motivate behavioural change, tailoring is used to apply the concepts of the transtheoretical model, and the health belief model. The intervention focuses on 3 core behaviours: controller medication adherence, rescue inhaler availability, and smoking cessation/reduction.	Online; individual	N/A	Students access programme using computers at participating schools	180 days given to complete all 4 sessions.	Yes	Yes
Klee et al.^33^	Webdia— patient‐designed do‐it‐yourself mobile device app	To evaluate the impact of a multidisciplinary intervention consisting of using Webdia, a patient‐designed mHealth app for smartphones, combined with an educational intervention by specialised nurses and regular insulin dose adaptation by diabetologists on metabolic control of T1DM, QoL and frequency of hypoglycemia in children. Webdia was developed by a father whose 10‐year‐old daughter was diagnosed with T1DM, with the aim of improving his daughter's autonomy and facilitate data exchange within the family.	Smartphone app; individual Initial face‐to‐face tutorial; individual Email; individual *Phone call; individual*	Initial tutorial given by a specialised nurse Unclear who made phone call after 1 month	NR	Initial tutorial: 45 min, one off Webdia—to use as often as possible for 3 months.	No	Yes
Lam et al.^34^	Internet‐based multimodal pain programme	To investigate the treatment effect of an internet‐based multimodal pain programme on chronic TMD pain and evaluate the feasibility of a larger randomised controlled trial. The intervention was based on cognitive behaviour therapy and self‐management principles and adapts face‐to‐face therapy to an online platform (software programme), designed to be used without guidance.	Online; individual Phone call; individual, at start‐up Workbook; individual *Email; individual* *Asynchronous chat; individual*	Dentist with A 2‐day training on internet‐based CBT provided support: for study adherence only	NR	The intended treatment duration was 7 weeks: 1 module/week; 40 min/module online plus time for homework assignments.	No	Yes
Linden et al.^35^	Person‐centred, web‐based support programme	To determine the effectiveness of a person‐centred, web‐based support intervention to be used during pregnancy and in early motherhood by women with T1DM. The intervention focused on strengthening autonomy and personal capacity, thereby optimising well‐being and self‐efficacy of diabetes management. Further, the study aimed to explore the use of the web‐based support. The intervention was based on person‐centred care, and designed to assist in decision‐making, to support self‐care and to facilitate peer contact.	Online; individual plus discussion forum (for peer support) text messages; individual (if needed)	N/A	NR	Early pregnancy to 6 months post birth. Had to log on at least twice to be adherent. No requirement regarding time spent logged onto system.	No	Yes
Middleton et al.^36^	Enhanced SMS text‐based support and reminder programme + standard care	To examine the effectiveness of an enhanced SMS text message–based support and reminder programme in improving clinic attendance, metabolic control, engagement in self‐management, and psychological health in a young‐onset T2DM cohort.	Text messages; individual	Automated *Study specific SMS portal allowing participants to send questions about diabetes and its management directly to the study team*	NR	First week: introductory message. First 2 months: 2 messages a week. Third month: 1 message a week Thereafter: 1 message a month. Messages sent at random times during business hours: Monday to Friday, 9:00 AM to 5:00 PM. Appointment reminders sent week before follow‐up.	Yes	NR
Whiteley et al.^37^	BattleViro: iPhone gaming adherence intervention with game related text messages	To examine the preliminary effects of BattleViro—a multilevel gaming intervention for iPhone—on ART adherence, viral load, and relevant knowledge and attitudes among youth living with HIV. The gaming app aimed to empower youth to improve adherence by increasing information, motivation, and behavioural skills. The intervention was based on Information Motivation and Behavioural Skills and used an asset model, to promoted self‐mastery and social support for adherence.	Smartphone gaming app; individual Text messages; individual	N/A	NR	14 weeks, as and when.	No	NR

*Note*: Italicised modes of delivery/intervention providers indicate an element reported as specific to the study rather than integral to the intervention itself.

Abbreviations: CBT, cognitive behavioural therapy; CF, cystic fibrosis; IBD, inflammatory bowel disease; mHealth apps, mobile health applications; N/A, not applicable; NR, not reported; QoL, quality of life; SM, self‐management; SMS, short message service; T1DM, type I diabetes mellitus; T2DM, type II diabetes mellitus; TMD, temporomandibular disorder; WebEase, Web Epilepsy Awareness, Support and Education.

**Table 3:** Delivery modes and components of interventions: For each section, these are presented in descending order of frequency of occurrence within interventions across studies.

	*Ayar* et al.^24^	Bell et al.^26^	*Chapman* et al.^27^	Dilorio et al.^28^	*Huang* et al.^30^	Joseph et al.^32^	Lam et al.^34^	Linden et al.^35^	Klee et al.^33^	Whiteley et al.^37^	*Balato* et al.^25^	*Middleton* et al.^36^	Hockenmeyer and Smyth^29^	*Hunt* et al.^31^	Number of studies
Intervention (columns)	‘*Youth diabetes’—web‐based diabetes education + standard medical care*	Narrative care messages	*Tailored digital intervention to change adherence‐related beliefs and barriers*	WebEase: theory‐based, interactive, internet‐based self‐management programme for people with epilepsy	*MD2Me— technology‐based disease management intervention*	Puff city: multimedia, web‐based asthma management programme	Internet‐based multimodal pain programme	Person‐centred, web‐based support programme	Webdia: Patient‐designed Do‐It‐Yourself Mobile Device App	Battleviro: iPhone gaming adherence intervention with game‐related text messages	*Text messages*	*Enhanced SMS text‐based support and reminder programme + standard care*	Self‐administered manual‐based stress management intervention	*Self‐help CBT book*	
Element (row)															
Condition	*T1DM*	T1DM	*IBD*	Epilepsy	*Chronic disease: IBD, CF, T1DM*	Asthma	Chronic TMD pain	T1DM	T1DM	HIV	*Psoriasis*	*T2DM*	Asthma	*IBD*	
**Intervention delivery modes**															
***Mode used as primary mode only across interventions***															
Web‐based (individual)	***X***	X	***X***	X	***X***	X	X	X							**8**
Mobile device app									X	X					**2**
***Mode used as both primary and secondary modes across interventions***															
Text message					***X***			X		X	***X*** a	***X*** a			**5**
Workbook/Booklet							X						X^a^	***X*** a	**3**
***Mode used as secondary mode only across interventions***															
Audiovisual recordings			***X***	X									X		**3**
Web‐based (group)	***X***							X							**2**
Phone call							X		X^b^						**2**
E‐mail							X^c^		X						**2**
Face‐to‐face (individual)									X						**1**
***Number of modes utilised for intervention***															
Combination of modes (≥2 modes)	***X***		***X***	X	***X***		X	X	X	X			X		**9**
Single mode		X				X					***X***	***X***		***X***	**5**
**Additional intervention deliverer (beyond self‐administration)**															
Healthcare team					***X***		X^c^		X			***X*** d			**4**
Automated			***X***		***X***							***X***			**3**
**Intervention components**															
**Behavioural change techniques (BCTs)**															
Reminders, prompts and cues (memory aids)	***X***		***X***	X	***X***			X			***X***	***X***			**7**
Recording and handling of data including diary			***X***	X			X	X	X						**5**
Problem solving			***X***		***X***		X						X		**4**
Action planning			***X***	X	***X***		X								**4**
Feedback				X		X	X^c^			X					**4**
Goal setting			***X***	X			X								**3**
Cognitive restructuring (Thought records)			***X***										X	***X***	**3**
Signposting to medical care team(s)			***X***		***X***							***X***			**3**
Signposting to other social support^e^			***X***		***X***							***X***			**3**
CBT components or exercises							X						X		**2**
Pros and cons			***X***	X											**2**
Reflect previous success			***X***				X								**2**
Signposting to additional resources			***X***					X							**2**
Time management							X								**1**
Brainstorming					***X***										**1**
Eliminating avoidance behaviours														***X***	**1**
Reflect on current behaviours				X											**1**
Behavioural experiments														***X***	**1**
Credible source			***X***												**1**
Information about health consequences			***X***												**1**
Pharmacological support			***X***												**1**
Direct contact with healthcare team					***X***										**1**
Restructuring of physical environment			***X***												**1**
Habit formation			***X***												**1**
Behavioural practice/rehearsal			***X***												**1**
Demonstration of the behaviour			***X***												**1**
**Number of BCTs**	**1**	**0**	**18**	**7**	**7**	**1**	**8**	**3**	**1**	**1**	**1**	**3**	**3**	**3**	
**Other components**															
Provision of stress/anxiety specific information	***X***			X	***X***		X					***X***	X	***X***	**7**
Materials designed in a simple format				X		X			X		***X***	***X***	X		**6**
Tailoring to individual			***X***	X	***X***	X						***X***			**5**
Quiz/poll questions	***X***			X						X				***X***	**4**
Disease‐specific examples and narratives		X		X	***X***									***X***	**4**
Relaxation							X						X	***X***	**3**
Blog/discussion forum	***X***			X				X							**3**
Techniques to improve lifestyle					***X***									***X***	**2**
Assessment				X			X								**2**
Writing or drawing exercises							X						X		**2**
Exercises (rehabilitation)							X								**1**
Monitoring devices										X					**1**
**Number of other components**	***3***	1	***1***	7	4	2	5	1	1	2	***1***	3	4	***5***	
**Total number of components**	**4**	**1**	**19**	**14**	**11**	**3**	**13**	**4**	**2**	**3**	**2**	**6**	**7**	**8**	

*Note*: If the author and column are **emboldened** italics, then the intervention was significantly more beneficial regarding outcomes of interest compared to the control.

Abbreviations: CBT, cognitive behavioural therapy; CF, cystic fibrosis; HCP, healthcare professional; IBD, inflammatory bowel disease; T1DM, type 1 diabetes mellites; T2DM, type 2 diabetes mellites; TMD, temporomandibular disorder; WebEase, Web Epilepsy Awareness, Support and Education.

^a^ Primary mode.

^b^ Intervention intended to be used without guidance, during the study only, the support provided (to aid study adherence).

^c^ While the intervention was intended to be used without guidance, during the study, support was provided by a dentist to aid study adherence: at the end of each module, individuals received a follow‐up phone call (email or asynchronous chat) to provide support and feedback.

^d^ Study‐team (including HCPs) accessible via study‐specific SMS portal could be sent questions re diabetes and its management.

^e^ Family, friends, support groups.

All but two included self‐management interventions that were self‐directed without any support. Of the two interventions which involved HCPs, neither provided regular, ongoing support. One included an HCP delivering an initial 45 min tutorial on installation and use of the mobile health application^33^ (HCP input related to study processes rather than intervention delivery); and the second utilised an HCP to respond to health concerns of individuals when activated via SMS algorithm^30^ (HCP contact separate from the main intervention, an optional extra available to the participants but not received routinely). Klee et al.^33^ along with two further studies^34^, ^36^ introduced study‐specific elements which our review team felt had the potential to impact study outcomes so were tentatively considered part of the interventions tested: Klee et al.^33^ included a single phone call to participants after 1 month to ensure there were no technical problems and to answer any questions. Given the lack of clarity regarding what these questions concerned, it was deemed possible that outcomes may be affected so was considered integral to the intervention; Lam et al.^34^ ^(^^p.3)^ involved a dentist ‘to support study adherence’, who had received 2‐day training on internet‐based cognitive behavioural therapy (CBT) to provide email, phone or asynchronous chat support to individuals with chronic temporomandibular pain disorder. While Middleton et al.^36^ introduced a study‐specific SMS portal where participants had the option to send questions about diabetes and its management to the study team which included healthcare professionals. When synthesising the findings, these specific intervention characteristics were included in a sensitivity analysis to establish what if any effect their inclusion/exclusion had on the overall strength of evidence (see below).

Detailed analysis of the 14 interventions reported, identified a total of 38 components (26 behavioural change techniques [BCT] and 12 other components) (Table 3). Interventions contained a median of five components (interquartile range [IQR]: 3–10.25, range 1–19), with all but one intervention^26^ utilising between 1 and 18 BCTs (median = 3, IQR: 1–6).

The majority of components were utilised in only one or two interventions (58%, *n* = 22, Table 3) with a median of two interventions using each component (IQR: 1–3.75, range 1–7). The most frequently used components were memory aids such as reminders and prompts (BCT) or the provision of stress or anxiety‐specific information (*n* = 7, 50% of interventions). Other components used by at least a quarter of interventions were materials designed in a simple format, that is, with the aim to be as accessible as possible to people with a range of reading and health literacy levels, for example, using pictures and simplified language (*n* = 6), tailoring to the individual and recording and handling of data, such as symptom and medication diaries, logging sleep quality, stress, and so forth (*n* = 5 each), problem‐solving (BCT), action planning (BCT), feedback (BCT), disease‐specific examples and narratives and quiz/poll questions (*n* = 4 each) (Table 3). All interventions utilised at least one of the aforementioned components.

Web‐based interventions did not differ from other types of interventions regarding the number of intervention components (median = 7.5, IQR: 4–13.25 vs. median = 4.5, IQR: 2.5–6.75, respectively; Mann–Whitney *U* = 15.5, *p* > .05), the involvement of additional intervention ‘deliverers’, that is, healthcare team or automated (38%, *n* = 3 of eight interventions vs. 33%, *n* = 2 of six interventions; *χ* ^2^_1,14_ = 0.87, *p* = .87), or whether a combination of delivery modes were used (75%, *n* = 6 of eight interventions vs. 50%, *n* = 3 of six interventions; *χ* ^2^_1,14_ = 0.93, *p* = .69). Web‐based interventions were similar in structure to those delivered by other modes.

Self‐management interventions that were significantly more beneficial compared to controls concerning the review's outcomes of interest showed similar modes of delivery (web‐based *n* = 3, other *n* = 6) compared to interventions where no difference was detected (web‐based *n* = 5, other *n* = 3; *χ* ^2^_1,14_ = 0.2187, *p* = .640). Furthermore, they were equally as likely to include additional intervention deliverers (38%, *n* = 3 of 8 interventions vs. 33%, *n* = 2 of six interventions; *χ* ^2^_1,14_ = 0.87, *p* = .87), and reported similar number of components (median = 7, IQR: 4.5–10.25 vs. median = 3.5, IQR: 2.75–8.5, respectively; Mann–Whitney *U* = 17, *p* > .05). Thus, intervention characteristics (i.e., delivery mode, additional intervention deliverers and number of components) did not appear to impact on whether an intervention was beneficial or not.

### 3.4 Methodological quality of included studies

**Figure 3:**
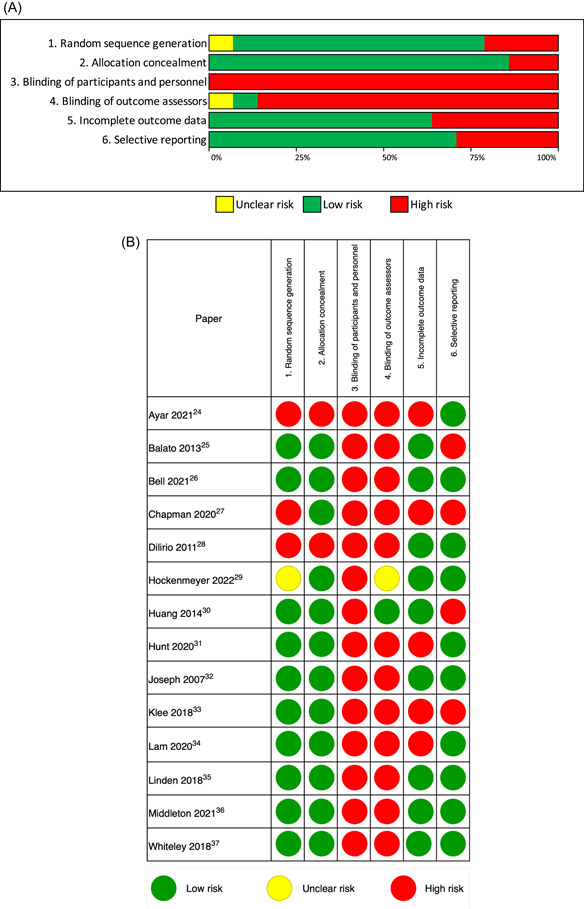
(A) Risk of bias aggregate. (B) The overall risk of bias for individual studies.

Across studies, risk of bias was generally low (>71% studies) for random sequence generation, allocation concealment and reporting (Figure 3A,B). Conversely, blinding of participants and personnel, and of outcome assessors were almost exclusively rated as high risk—a consequence of the nature of the intervention, meaning blinding is not achievable. Five studies (36%) had high or unbalanced rates of attrition and were therefore identified as having a high risk of bias for incomplete outcome data (Figure 3B).

### 3.5 Overall certainty of the evidence

The overall certainty of the evidence for individual intervention characteristics was predominantly low (*n* = 36) or very low (*n* = 14), with only one element (single mode of delivery) rated as moderate (Table 4; detailed in Supporting Information: File 3). These low levels of certainty were largely driven by concerns regarding the risk of bias and applicability reported for more than 90% of components, while concerns with the number of studies, consistency, and precision ranged from 41% to 53% of components.

**Table 4:** Overall certainty of evidence for intervention characteristics. Characteristics in italics involved in sensitivity analysis, where studies where the characteristic was not intended to be part of the self‐directed intervention i.e. was specific to study only, were excluded and overall certainty of evidence reassessed (any changes to individual concerns and overall certainty are indicated).

Intervention characteristic	*n* studies	Concerns regarding				OVERALL certainty
		Risk of bias	Consistency	Precision	Applicability	
**Intervention delivery modes:**						
***Mode used as primary mode only across interventions***						
Web‐based (individual)	8	**Concern**	**Concern**	**No concern**	**Concern**	**Low**
Mobile device app	2	**Concern**	**Concern**	**Concern**	**No concern**	**Very low**
***Mode used as both primary and secondary modes across interventions***						
Workbook/booklet	3	**Concern**	**Concern**	**Concern**	**Concern**	**Very low**
Text message	5	**No concern**	**No concern**	**Concern**	**Concern**	**Low**
***Mode used as secondary mode only across interventions***						
Web‐based (group)	2	**Concern**	**No concern**	**No concern**	**Concern**	**Low**
*Phone call*	2	**Concern**	**Concern**	**Concern**	**Concern**	**Very low**
*Email*	2	**Concern**	**Concern**	**Concern**	**Concern**	**Very low**
Audio‐visual recordings	3	**Concern**	**Concern**	**No concern**	**Concern**	**Low**
Face‐to‐Face (individual)	1	**Concern**	**Concern**	**Concern**	**Concern**	**Very low**
***Number of modes utilised for intervention***						
Single mode	5	**No concern**	**No concern**	**No concern**	**Concern**	**Moderate**
Combination of modes ( ≥ 2 modes)	9	**Concern**	**Concern**	**Concern**	**Concern**	**Very low**
**Additional intervention deliverer (beyond self‐administration)**						
*Health care team/professionals*	4	**Concern**	**No concern**	**Concern**	**Concern** 1	**Low**
Automated	3	**Concern**	**No concern**	**Concern**	**Concern**	**Low**
**Behaviour‐change techniques**						
CBT components or exercises	2	**Concern**	**Concern**	**Concern**	**Concern**	**Very low**
Problem solving	4	**Concern**	**No concern**	**Concern**	**Concern**	**Low**
Goal setting	3	**Concern**	**Concern**	**No concern**	**Concern**	**Low**
Action planning	4	**Concern**	**No concern**	**No concern**	**Concern**	**Low**
Time management	1	**Concern**	**Concern**	**Concern**	**Concern**	**Very low**
Brainstorming	1	**Concern**	**No concern**	**Concern**	**No concern**	**Low**
Pros and cons	2	**Concern**	**No concern**	**No concern**	**Concern**	**Low**
Eliminating avoidance behaviours	1	**Concern**	**No concern**	**No concern**	**Concern**	**Low**
Cognitive restructuring (Thought records)	3	**Concern**	**No concern**	**No concern**	**Concern**	**Low**
Reflect on current behaviours	1	**Concern**	**Concern**	**No concern**	**Concern**	**Very low**
Reflect previous success	2	**Concern**	**No concern**	**No concern**	**Concern**	**Low**
Reminders, prompts & cues (Memory aids)	7	**Concern**	**No concern**	**Concern**	**Concern**	**Low**
*Feedback*	4	**Concern** 1	**Concern**	**No concern**	**Concern**	**Low**
Recording and handling of data including diary	5	**Concern**	**Concern**	**No concern**	**Concern**	**Low**
Behavioural experiments	1	**Concern**	**No concern**	**No concern**	**Concern**	**Low**
Credible source	1	**Concern**	**No concern**	**No concern**	**Concern**	**Low**
Information about health consequences	1	**Concern**	**No concern**	**No concern**	**Concern**	**Low**
Pharmacological support	1	**Concern**	**No concern**	**No concern**	**Concern**	**Low**
Direct contact with health care team	1	**Concern**	**No concern**	**Concern**	**No concern**	**Low**
Signposting to medical care team(s)	3	**Concern**	**No concern**	**Concern**	**Concern**	**Low**
Signposting to other social support	3	**Concern**	**No concern**	**Concern**	**Concern**	**Low**
Signposting to additional resources	2	**Concern**	**No concern**	**No concern**	**Concern**	**Low**
Restructuring of physical environment	1	**Concern**	**No concern**	**No concern**	**Concern**	**Low**
Habit formation	1	**Concern**	**No concern**	**No concern**	**Concern**	**Low**
Behavioural practice/rehearsal	1	**Concern**	**No concern**	**No concern**	**Concern**	**Low**
Demonstration of the behaviour	1	**Concern**	**No concern**	**No concern**	**Concern**	**Low**
**Intervention components**						
Provision of stress/anxiety specific information	7	**Concern**	**No concern**	**Concern**	**Concern**	**Low**
Materials designed in a simple format	6	**Concern**	**Concern**	**Concern**	**Concern**	**Very low**
Tailoring to individual	5	**Concern**	**No concern**	**No concern**	**Concern**	**Low**
Disease specific examples and narratives	4	**Concern**	**No concern**	**No concern**	**Concern**	**Low**
Blog/Discussion forum	3	**Concern**	**Concern**	**No concern**	**Concern**	**Low**
Assessment	2	**Concern**	**Concern**	**No concern**	**Concern**	**Very low**
Quiz/Poll questions	4	**Concern**	**No concern**	**No concern**	**Concern**	**Low**
Writing or drawing exercises	2	**Concern**	**Concern**	**Concern**	**Concern**	**Very low**
Exercises (rehabilitation)	1	**Concern**	**Concern**	**Concern**	**Concern**	**Very low**
Relaxation	3	**Concern**	**Concern**	**Concern**	**Concern**	**Very low**
Techniques to improve lifestyle	2	**Concern**	**No concern**	**No concern**	**No concern**	**Low**
Electronic pill monitoring device	1	**No concern**	**Concern**	**Concern**	**No concern**	**Low**
**Concern** ***n*** =	27	48	21	23	46	
**No concern** ***n =***	24	3	30	28	5	
					**High** ***n*** **=**	0
					**Moderate** ***n*** **=**	1
					**Low** ***n*** **=**	36
					**Very low** ***n*** **=**	14

^1^ sensitivity analysis led to change from ‘concern’ to ‘no concern’

Sensitivity analyses showed little change in the overall certainty of the evidence. First, excluding studies with study‐specific intervention characteristics that were not part of the intended intervention,^32^, ^33^, ^35^ found no change in overall certainty of the evidence for any of the four intervention characteristics affected, that is, phone call, email, healthcare team, feedback (Supporting Information: File 3). Reanalysis excluding studies with active controls^30^, ^31^, ^34^ affected 34 intervention characteristics (Supporting Information: File 4); however, for the majority (*n* = 23), there was no change in overall certainty of evidence. Seven intervention components disappeared: time management; brainstorming; eliminating avoidance behaviours; behavioural experiments; direct contact with the healthcare team; exercise (rehabilitation) and techniques to improve lifestyle. While three intervention characteristics were downgraded from low to very low certainty of evidence (delivery by healthcare team/professionals; disease‐specific examples and narratives; quiz/poll questions), and one upgraded from very low to low certainty of evidence (workbook/booklet as the primary mode of delivery).

## 4 DISCUSSION

This rapid review identified a paucity of interventions that tested self‐directed, self‐management interventions aiming, in whole or part, to prevent or address distress, mental health needs, and wellbeing of young people with physical LTCs. While 9 of the 14 studies included were reported to be beneficial, only 6 had a significant treatment benefit in reducing young people's distress or improving their wellbeing and self‐efficacy. However, it should be noted that only 3 of the 14 studies explicitly identified distress, wellbeing or self‐efficacy as their primary outcome. Four modes of intervention delivery were identified across the 14 studies—websites, smartphone applications, text messages and workbooks; and within these, 38 individual components were included across the interventions (see Table 3). However, modes of delivery and intervention components were found to be similar between beneficial and nonbeneficial interventions, making it difficult to identify which modes or components resulted in positive or outcomes or otherwise. Reasons for this finding may be the differences in the health conditions focused on, the variation in the combination of components across interventions, and the different ways in which these components were operationalised.

### 4.1 Comparison with previous literature

These review findings show similarity with other literature, including systematic reviews of self‐management interventions in other populations. In a reflective article on self‐management of LTCs, Trappenburg et al. argue that trials of self‐management interventions are characterised by ‘large heterogeneity in programme characteristics, such as mode, dose, intensity and delivery of self‐management programmes, resulting in a large variance in effect sizes’, as well as targeting ‘heterogeneous patient populations using varied outcome measures’.^38^ ^(^^p.136)^ As a result, they reached the conclusion that understanding the impact of individual intervention components and modes of delivery on trial outcomes is extremely challenging.

A similar conclusion was reached by Miller et al.^39^ in a systematic review of self‐management interventions delivered to people with multimorbidity. They too identified wide variation in delivery methods and components included in interventions, with the most frequently used component being education to improve participants' understanding of their conditions. Depression was the most common outcome measured across the studies, as both a disease‐specific and general outcome. The authors conclude that variation in self‐management programmes ‘limits conclusive evidence for which components are recommended to improve self‐management in individuals with multimorbidity’.^39^ ^(^^p.948)^ Focusing on a more specific population, Goodman et al. explored the effect of self‐management interventions tested in trials on quality of life, self‐management skills and self‐efficacy in people with a bowel stoma.^40^ They focused on the behaviour change techniques (BCTs) included within these interventions, identifying BCTs such as receiving information from credible sources, instruction on how to perform a particular behaviour and monitoring of behaviour. However, they similarly reported that the heterogeneity of interventions and outcomes measured meant they were unable to determine which of the BCTs that were used influenced participant outcomes.

### 4.2 Interpretation of results by people with lived experience (PPIE)

In discussing the review findings with our PPIE group, they highlighted the lack of detailed information about the interventions included in the review, which they felt made it difficult to assess whether trial outcomes were a result of intervention components and modes of delivery or the way in which these were operationalised. For instance, in relation to educational materials, they reflected that it was not possible to assess the quality of the information and educational content provided. The group also discussed the difficulty in evaluating interventions that combined a range of different components or modes of delivery, highlighting the challenge in identifying which of these modes/components may have had the most influence on participant outcomes. For instance, in relation to Ayar et al., who combined multimedia condition management tutorials, a peer group chat room, and quizzes targeted towards people with type 1 diabetes, it was speculated that positive outcomes in young people's wellbeing may have come mainly from the peer support received through the chat room.^24^

Members of the PPIE group drew attention to the trial follow‐up period reported in the studies. It was felt that interventions that were not beneficial, this could be explained by the short follow‐up period. The group identified Bell et al., who tested an intervention for type 1 diabetes comprising narrative online messages based on the lived experience of the condition, measuring outcomes on the same day participants accessed these messages.^26^ The PPIE group felt that this intervention had promise, but that trial participants were unlikely to have had time for the intervention content to ‘sink in’. From their own experiences of mood fluctuating over time, the group felt that 12 weeks would be a minimum time period to see an impact on distress, mental health or wellbeing. The group's arguments align with those expressed elsewhere. Gardner et al. propose the need for ‘realistic expectations’ regarding emotional or behavioural change and that it can take around 10 weeks to develop ‘daily habits’.^41^

When discussing lessons from the findings for the development of the future Stoma Support intervention, the group felt that self‐management workbooks may be beneficial, suggesting value in being able to work through information at their own pace, in a logical order. A workbook intervention was used in two studies in the review,^29^, ^31^ and was found to have a positive impact on psychological distress in Hunt et al.,^31^ who tested an intervention for young people with IBD. The group suggested this could be an online or physical workbook or a choice between the two. The group felt that a website or smartphone app could be beneficial for managing distress related to stoma surgery but did not feel that unsolicited text messages would be beneficial. Text messages were used in four interventions included in the review^25^, ^30^, ^36^, ^37^; three of which were found to be beneficial, though two of these combined text messages with other modes of delivery. Members of our PPIE group also suggested that receiving text messages may become irritating and are likely to be deleted, though they were more positive about receiving personalised rather than generic messages. Personalised messages were used by Chapman et al. in an intervention for individuals with IBD (though these were online rather than text messages), which had positive outcomes for psychological distress and wellbeing.^27^ The group felt that this tailoring of messages to focus on things important to participants might explain the positive outcomes of this trial. Overall, the ability to individualise and for interventions to be self‐directed was seen as positive.

An intervention component that was highlighted as potentially beneficial was CBT techniques, which were explicitly incorporated in two of the interventions in the review.^29^, ^31^ Members of the PPIE group felt that for people with an IBD stoma, the focus on psychological therapies and targeting emotions would be particularly beneficial, alongside being able to learn and use these techniques as part of a self‐directed self‐management intervention.

### 4.3 Implications for future design and testing of self‐directed self‐management interventions

A key finding in this rapid review is the paucity of interventions directly targeting distress and wellbeing in young people with physical LTCs. Six of the included studies targeted condition‐specific outcomes, with mental health, wellbeing and self‐efficacy being secondary outcomes, and five did not clearly specify the difference between their primary and secondary outcomes. Only 3 of the 14 studies explicitly targeted mental health, wellbeing and/or self‐efficacy as a primary outcome. Given the well‐established psychological impact of long‐term physical illness on mental health and wellbeing in this population,^42^ there is clearly a need for the development of high‐quality, evidence‐based self‐management interventions for this population that target mental health and wellbeing as primary outcomes.

In the review, we identified a lack of consistency in the way self‐management interventions for young people with physical LTCs have been developed and evaluated, which may be in part due to the variation in conditions targeted. This presents challenges in developing a core body of evidence to guide the design of future interventions for this population. One way of addressing this can be through more detailed and transparent reporting of intervention design, content and delivery which could help with future intervention design by allowing a more thorough assessment of how intervention components are operationalised and delivered. This would allow for a clearer picture of whether individual components led to improved outcomes, or whether this was down to how they were designed and delivered. This was a point raised in the discussion by our PPIE group, which has also been expressed elsewhere in the literature.^17^ Linked to this, and in line with UK Medical Research Council guidance,^43^ there is the need for robust process evaluations within trials of self‐management interventions, including qualitative research that explores participants' experiences and perceptions of which components and modes of delivery were acceptable and felt to be most beneficial.

In relation to intervention evaluation, there is a need for the development of a core outcome set for measuring mental health and wellbeing outcomes in this population, that would allow for comparison in evaluating trial results. This would be a useful area for future research. Consistency is also needed in the time period for measuring outcomes, another point that was highlighted by our PPIE group. We would suggest the incorporation of PPIE input in deciding follow‐up periods; our PPIE group highlighted 12 weeks as a minimum time period in their experience, to bring about meaningful change.

### 4.4 Strengths and limitations

A clear strength of this review was the inclusion of PPIE members in helping to interpret the review findings and drawing out the key implications. We were able to harness the group's experiential expertise to identify key messages from the findings, as well as ensure that the review findings were ‘relevant and meaningful’ to those from the population of interest.^44^ ^(^^p.1)^ A limitation of the study is that the shorter timeframe for this rapid review when compared to a traditional systematic review meant that we were unable to include articles not written in English, and as a result may have excluded potentially relevant articles.

## 5 CONCLUSION

We have found a lack of studies testing self‐directed self‐management interventions for young people with physical LTCs that directly target mental health and wellbeing outcomes. In those that were identified, it was not possible to conclude which components or modes of delivery resulted in beneficial outcomes. However, significant incorporation of PPIE input enabled the development of future recommendations, including the need for more detailed and transparent reporting of self‐management intervention design, content and delivery and robust process evaluation to enable a better understanding of which components and modes of delivery brought about change. Additionally, there is a need for a core outcome set for measuring mental health and wellbeing outcomes in relation to self‐management interventions in this population, and consistency in follow‐up periods. These findings can have implications for future research and intervention design and will also inform the next stages of the Stoma Support Study in which we will work with young people with a stoma and healthcare professionals to co‐design the content and format of a self‐management intervention for young people with an IBD stoma.

## AUTHOR CONTRIBUTIONS

Adam D. Farmer, Benjamin Saunders, Carolyn A. Chew‐Graham, Kay Polidano, Lucy Bray, Megan McDermott‐Hughes and Nadia Corp contributed to the original conception of the work. Benjamin Saunders, Lucy Bray and Nadia Corp completed literature searches, screening, data extraction and appraisal. Nadia Corp led the synthesis with oversight from Benjamin Saunders. All authors contributed to the drafting of this manuscript and agreed to the final version.

## CONFLICT OF INTEREST STATEMENT

Carolyn A. Chew‐Graham has a working role within the *Health Expectations Journal*. The remaining authors declare no conflict of interest.

## Supporting information

Supporting information.

Supporting information.

Supporting information.

Supporting information.

## Data Availability

All data relevant to the study are included in the article or uploaded as Supporting Information.

## References

[1] Read JR , Sharpe L , Modini M , Dear BF . Multimorbidity and depression: a systematic review and meta‐analysis. J Affect Disord. 2017;221:36‐46. 10.1016/j.jad.2017.06.009 28628766

[2] Geraghty AW , Santer M , Williams S , et al. ‘You feel like your whole world is caving in’: a qualitative study of primary care patients’ conceptualisations of emotional distress. Health. 2017;21(3):295‐315. 10.1177/1363459316674786 28177273 PMC5439536

[3] Balfe M . The body projects of university students with type 1 diabetes. Qual Health Res. 2009;19(1):128‐139. 10.1177/1049732308328052 19029244

[4] Saunders B . ‘It seems like you're going around in circles’: recurrent biographical disruption constructed through the past, present and anticipated future in the narratives of young adults with inflammatory bowel disease. Sociol Health Illn. 2017;39(5):726‐740. 10.1111/1467-9566.12561 28425115

[5] Bronckers IMGJ , Paller AS , van Geel MJ , van de Kerkhof PCM , Seyger MMB . Psoriasis in children and adolescents: diagnosis, management and comorbidities. Pediatric Drugs. 2015;17(5):373‐384. 10.1007/s40272-015-0137-1 26072040 PMC4744260

[6] Polidano K , Chew‐Graham CA , Farmer AD , Saunders B . Access to psychological support for young people following stoma surgery: exploring patients' and clinicians' perspectives. Qual Health Res. 2021;31(3):535‐549. 10.1177/1049732320972338 33228473 PMC7802047

[7] Panagioti M , Richardson G , Small N , et al. Self‐management support interventions to reduce health care utilisation without compromising outcomes: a systematic review and meta‐analysis. BMC Health Serv Res. 2014;14:356. 10.1186/1472-6963-14-356 25164529 PMC4177163

[8] Royal College of Nursing (RCN) . Self‐care. 2021. Accessed September 29, 2021. https://www.rcn.org.uk/clinicaltopics/public-health/self-care

[9] National Health Service (NHS) . The NHS long term plan. BMJ. 2019;364:l84. 30617185 10.1136/bmj.l84 PMC6350418

[10] Sattoe JNT , Bal MI , Roelofs PDDM , Bal R , Miedema HS , van Staa A . Self‐management interventions for young people with chronic conditions: a systematic overview. Patient Educ Couns. 2015;98(6):704‐715. 10.1016/j.pec.2015.03.004 25819373

[11] Department of Health and Social Care . Wellbeing and Why it Matters to Health. Department of Health and Social Care; 2014.

[12] Cramm JM , Strating MMH , Roebroeck ME , Nieboer AP . The importance of general self‐efficacy for the quality of life of adolescents with chronic conditions. Soc Indic Res. 2013;113(1):551‐561. 10.1007/s11205-012-0110-0 23874059 PMC3696170

[13] Polidano K , Chew‐Graham CA , Bartlam B , Farmer AD , Saunders B . Embracing a ‘new normal’: the construction of biographical renewal in young adults' narratives of living with a stoma. Sociol Health Illn. 2020;42(2):342‐358. 10.1111/1467-9566.13005 31562644

[14] Boote J , Baird W , Sutton A . Involving the public in systematic reviews: a narrative review of organizational approaches and eight case examples. J Comp Eff Res. 2012;1(5):409‐420. 10.2217/cer.12.46 24236418

[15] Oliver K , Rees R , Brady LM , Kavanagh J , Oliver S , Thomas J . Broadening public participation in systematic reviews: a case example involving young people in two configurative reviews. Res Synthesis Methods. 2015;6(2):206‐217. 10.1002/jrsm.1145 PMC5008219 26099487

[16] Pollock A , Campbell P , Struthers C , et al. Development of the ACTIVE framework to describe stakeholder involvement in systematic reviews. J Health Serv Res Policy. 2019;24(4):245‐255. 10.1177/1355819619841647 30997859

[17] Harris J , Croot L , Thompson J , Springett J . How stakeholder participation can contribute to systematic reviews of complex interventions. J Epidemiol Community Health. 2016;70(2):207‐214. 10.1136/jech-2015-205701 26475921 PMC4752615

[18] Garritty C , Gartlehner G , Nussbaumer‐Streit B , et al. Cochrane Rapid Reviews Methods Group offers evidence‐informed guidance to conduct rapid reviews. J Clin Epidemiol. 2021;130:13‐22. 10.1016/j.jclinepi.2020.10.007 33068715 PMC7557165

[19] Page MJ , McKenzie JE , Bossuyt PM , et al. The PRISMA 2020 statement: an updated guideline for reporting systematic reviews. BMJ. 2021;372:n71. 10.1136/bmj.n71 33782057 PMC8005924

[20] Campbell M , McKenzie JE , Sowden A , et al. Synthesis Without Meta‐analysis (SWiM) in systematic reviews: reporting guideline. BMJ. 2020;368:l6890. 10.1136/bmj.l6890 31948937 PMC7190266

[21] Hoffmann TC , Glasziou PP , Boutron I , et al. Better reporting of interventions: template for intervention description and replication (TIDieR) checklist and guide. BMJ. 2014;348:g1687. 10.1136/bmj.g1687 24609605

[22] Higgins JPT , Altman DG , Gotzsche PC , et al. The Cochrane Collaboration's tool for assessing risk of bias in randomised trials. BMJ. 2011;343:d5928. 10.1136/bmj.d5928 22008217 PMC3196245

[23] Guyatt GH , Oxman AD , Vist GE , et al. GRADE: an emerging consensus on rating quality of evidence and strength of recommendations. BMJ. 2008;336(7650):924‐926. 10.1136/bmj.39489.470347.AD 18436948 PMC2335261

[24] Ayar D , Öztürk C , Grey M . The effect of web‐based diabetes education on the metabolic control, self‐efficacy and quality of life of adolescents with type 1 diabetes mellitus in Turkey. J Pediatr Res. 2021;8(2):131‐138. 10.4274/jpr.galenos.2020.61214

[25] Balato N , Megna M , Di Costanzo L , Balato A , Ayala F . Educational and motivational support service: a pilot study for mobile‐phone‐based interventions in patients with psoriasis. Br J Dermatol. 2013;168(1):201‐205. 10.1111/j.1365-2133.2012.11205.x 23240729

[26] Bell T , Noar SM , Lazard AJ . Narrative vs. standard of care messages: testing how communication can positively influence adolescents with type 1 diabetes. J Health Commun. 2021;26:626‐635. 10.1080/10810730.2021.1985657 34649469

[27] Chapman S , Sibelli A , St‐Clair Jones A , Forbes A , Chater A , Horne R . Personalised adherence support for maintenance treatment of inflammatory bowel disease: a tailored digital intervention to change adherence‐related beliefs and barriers. J Crohn's Colitis. 2020;14(10):1394‐1404. 10.1093/ecco-jcc/jjz034 32379303

[28] DiIorio C , Bamps Y , Walker ER , Escoffery C . Results of a research study evaluating WebEase, an online epilepsy self‐management program. Epilepsy Behav. 2011;22(3):469‐474. 10.1016/j.yebeh.2011.07.030 21889413

[29] Hockemeyer J , Smyth J . Evaluating the feasibility and efficacy of a self‐administered manual‐based stress management intervention for individuals with asthma: results from a controlled study. Behav Med. 2002;27(4):161‐172. 10.1080/08964280209596041 12165970

[30] Huang JS , Terrones L , Tompane T , et al. Preparing adolescents with chronic disease for transition to adult care: a technology program. Pediatrics. 2014;133(6):e1639‐e1646. 10.1542/peds.2013-2830 24843066 PMC4035589

[31] Hunt MG , Loftus P , Accardo M , Keenan M , Cohen L , Osterman MT . Self‐help cognitive behavioral therapy improves health‐related quality of life for inflammatory bowel disease patients: a randomized controlled effectiveness trial. J Clin Psychol Med Settings. 2020;27(3):467‐479. 10.1007/s10880-019-09621-7 31025253

[32] Joseph CLM , Peterson E , Havstad S , et al. A web‐based, tailored asthma management program for urban African‐American high school students. Am J Respir Crit Care Med. 2007;175(9):888‐895. 10.1164/rccm.200608-1244OC 17290041 PMC1899296

[33] Klee P , Bussien C , Castellsague M , et al. An intervention by a patient‐designed do‐it‐yourself mobile device app reduces HbA1c in children and adolescents with type 1 diabetes: a randomized double‐crossover study. Diabetes Technol Ther. 2018;20(12):797‐805. 10.1089/dia.2018.0255 30403495

[34] Lam J , Svensson P , Alstergren P . Internet‐based multimodal pain program with telephone support for adults with chronic temporomandibular disorder pain: randomized controlled pilot trial. J Med Internet Res. 2020;22(10):e22326. 10.2196/22326 33048053 PMC7592067

[35] Linden K , Berg M , Adolfsson A , Sparud‐Lundin C . Person‐centred, web‐based support in pregnancy and early motherhood for women with type 1 diabetes mellitus: a randomized controlled trial. Diabetic Med. 2018;35(2):232‐241. 10.1111/dme.13552 29171071 PMC5814869

[36] Middleton T , Constantino M , McGill M , et al. An enhanced SMS text message‐based support and reminder program for young adults with type 2 diabetes (TEXT2U): randomized controlled trial. J Med Internet Res. 2021;23:e27263. 10.2196/27263 34524102 PMC8569538

[37] Whiteley L , Brown LK , Mena L , Craker L , Arnold T . Enhancing health among youth living with HIV using an iPhone game. AIDS Care. 2018;30(sup4):21‐33. 10.1080/09540121.2018.1503224 30626196 PMC6422754

[38] Trappenburg J , Jonkman N , Jaarsma T , et al. Self‐management: one size does not fit all. Patient Educ Couns. 2013;92(1):134‐137. 10.1016/j.pec.2013.02.009 23499381

[39] Miller JJ , Pozehl BJ , Alonso W , Schmaderer M , Eisenhauer C . Intervention components targeting self‐management in individuals with multiple chronic conditions: an integrative review. West J Nurs Res. 2020;42(11):948‐962. 10.1177/0193945920902146 32075541

[40] Goodman W , Allsop M , Downing A , et al. A systematic review and meta‐analysis of the effectiveness of self‐management interventions in people with a stoma. J Adv Nurs. 2022;78(3):722‐738. 10.1111/jan.15085 34708416

[41] Gardner B , Lally P , Wardle J . Making health habitual: the psychology of ‘habit‐formation’ and general practice. Br J Gen Pract. 2012;62(605):664‐666. 10.3399/bjgp12X659466 23211256 PMC3505409

[42] Colver A , McConachie H , Le Couteur A , et al. A longitudinal, observational study of the features of transitional healthcare associated with better outcomes for young people with long‐term conditions. BMC Med. 2018;16(1):111. 10.1186/s12916-018-1102-y 30032726 PMC6055340

[43] Skivington K , Matthews L , Simpson SA , et al. A new framework for developing and evaluating complex interventions: update of Medical Research Council guidance. BMJ. 2021;374:n2061. 10.1136/bmj.n2061 34593508 PMC8482308

[44] Pollock A , Campbell F , Synnot A , Smith M , Morley R. Patient and public involvement in systematic reviews. GIN Public Toolkit: Patient and Public Involvement in Guidelines. Guidelines International Network; 2021. https://g-i-n.net/wp-content/uploads/2023/07/Toolkit-combined.pdf

