# Supplementary material for: Self‐directed self‐management interventions to prevent or address distress in young people with long‐term physical conditions: A rapid review
Source: Health Expect. 2023 Aug 21;26(6):2164–90. doi: 10.1111/hex.13845 (PMC10632640; doi:10.1111/hex.13845)
Supplement: Supplementary file 1 — Supporting information. [file HEX-26--s002.docx]

**Supplementary file 1: Search strategies**

# **OVID databases**

The following table is an explanation of the symbols used in the OVID search strategies below.

/ indicates an index term (MeSH heading).

exp before an index term indicates that all subheadings were selected.

.ab. indicates a search for a term in the abstract.

.pt. indicates a search for term in publication type.

.sh. indicates a search for a term in subject heading.

.ti. indicates a search for a term in the title.

.ti,ab,kf. indicates a search for a term in title/abstract/word(s) in keyword [MEDLINE].

.ti,ab,kw. indicates a search for a term in title/abstract/word(s) in keyword [EMBASE, Cochrane].

.ti,ab. indicates a search for a term in title/abstract/word(s) in keyword [HMIC].

.tw. indicates a search for a term in the title/abstract.

*[*n*] at the end of a term indicates that this term has been truncated [by *n* character(s)].

adj indicates a search for two terms where they appear adjacent to each another.

adj*n* indicates a search for two terms where they appear within *n* words of each another.

# The mandated wild card character stands for one character within a word or at the end of a word.

? The optional wild card character stands for zero or one character within a word or at the end of a word.

# **MEDLINE (OvidSP)**

#

| \| 1 \| self care/ \| \| --- \| --- \| \| 2 \| self management/ \| \| 3 \| self medication/ \| \| 4 \| self car*.ti,ab,kf. \| \| 5 \| self help.ti,ab,kf. \| \| 6 \| self guided.ti,ab,kf. \| \| 7 \| self directed.ti,ab,kf. \| \| 8 \| self manag*.ti,ab,kf. \| \| 9 \| (patient* adj3 directed).ti,ab,kf. \| \| 10 \| (patient* adj3 guided).ti,ab,kf. \| \| 11 \| self improvement.ti,ab,kf. \| \| 12 \| or/1-11 \| \| 13 \| exp Chronic Disease/ \| \| 14 \| (chronic* adj3 (condition* or disease* or disorder* or illness*)).ti,ab,kf. \| \| 15 \| (long term adj3 (condition* or disease* or disorder or illness*)).ti,ab,kf. \| \| 16 \| (longterm adj3 (condition* or disease* or disorder or illness*)).ti,ab,kf. \| \| 17 \| (long standing adj3 (condition* or disease* or disorder or illness*)).ti,ab,kf. \| \| 18 \| (longstanding adj3 (condition* or disease* or disorder or illness*)).ti,ab,kf. \| \| 19 \| (degenerative adj3 (condition* or disease* or disorder or illness*)).ti,ab,kf. \| \| 20 \| (end stage adj3 (condition* or disease* or disorder or illness*)).ti,ab,kf. \| \| 21 \| (persistent adj3 (condition* or disease* or disorder* or illness*)).ti,ab,kf. \| \| 22 \| LTC*.ti,ab,kf. \| \| 23 \| chronic.hw. [subject heading word e.g chronic hepatitis] \| \| 24 \| exp epilepsy/ \| \| 25 \| exp diabetes mellitus/ \| \| 26 \| exp asthma/ \| \| 27 \| epilep*.ti,ab,kf. \| \| 28 \| diabet*.ti,ab,kf. \| \| 29 \| asthma*.ti,ab,kf. \| \| 30 \| exp Inflammatory Bowel Diseases/ \| \| 31 \| inflammatory bowel.ti,ab,kf. \| \| 32 \| IBD.ti,ab,kf. \| \| 33 \| ulcerative colitis.ti,ab,kf. \| \| 34 \| crohn*.ti,ab,kf. \| \| 35 \| stoma*1.ti,ab,kf. \| \| 36 \| exp Enterostomy/ \| \| 37 \| enterostom*.ti,ab,kf. \| \| 38 \| ileostom*.ti,ab,kf. \| \| 39 \| c?ecostom*.ti,ab,kf. \| \| 40 \| colostom*.ti,ab,kf. \| \| 41 \| duodenostom*.ti,ab,kf. \| \| 42 \| jejunostom*.ti,ab,kf. \| \| 43 \| or/13-42 \| \| 44 \| (young adj3 (adult* or person or people or wom#n or m#n)).ti,ab,kf. \| \| 45 \| (early adj3 adult*).ti,ab,kf. \| \| 46 \| (emerg* adj3 adult*).ti,ab,kf. \| \| 47 \| adolescen*.ti,ab,kf. \| \| 48 \| juvenile*.ti,ab,kf. \| \| 49 \| teen*.ti,ab,kf. \| \| 50 \| Young Adult/ \| \| 51 \| Adolescent/ [defined as 13-18 years old] \| \| 52 \| youth*1.ti,ab,kf. \| \| 53 \| or/44-52 \| \| 54 \| randomized controlled trial.pt. [rows 49-55 based on Cochrane Highly Sensitive Search Strategy: sensitivity- and precision-maximizing version (2008 revision)] \| \| 55 \| controlled clinical trial.pt. \| \| 56 \| randomi#ed.ab. [modified to pick up 's' and 'z' variants] \| \| 57 \| placebo.ab. \| \| 58 \| clinical trials as topic.sh. \| \| 59 \| randomly.ab. \| \| 60 \| trial.ti. \| \| 61 \| exp Cohort Studies/ \| \| 62 \| controlled before-after studies/ \| \| 63 \| historically controlled study/ \| \| 64 \| interrupted time series analysis/ \| \| 65 \| cohort.ti,ab,kf. \| \| 66 \| (before adj2 after).ti,ab,kf. \| \| 67 \| (pre adj2 post).ti,ab,kf. \| \| 68 \| (pretest adj2 posttest).ti,ab,kf. \| \| 69 \| (pre test adj2 post test).ti,ab,kf. \| \| 70 \| control*.ti,ab,kf. \| \| 71 \| time series.ti,ab,kf. \| \| 72 \| or/54-71 \| \| 73 \| 12 and 43 and 53 and 72 \| \| 74 \| Mental Health/ \| \| 75 \| Psychological Distress/ \| \| 76 \| exp Stress, Psychological/ \| \| 77 \| Depression/ \| \| 78 \| anxiety/ \| \| 79 \| depress*.ti,ab,kf. \| \| 80 \| distress*.ti,ab,kf. \| \| 81 \| stress*.ti,ab,kf. \| \| 82 \| low mood.ti,ab,kf. \| \| 83 \| (anxiet* or anxious*).ti,ab,kf. \| \| 84 \| (mental adj2 health).ti,ab,kf. \| \| 85 \| life satisfaction.ti,ab,kf. \| \| 86 \| "Quality of Life"/ \| \| 87 \| (quality adj2 life).ti,ab,kf. \| \| 88 \| (QoL or HRQoL).ti,ab,kf. \| \| 89 \| (well being or wellbeing).ti,ab,kf. \| \| 90 \| or/74-89 \| \| 91 \| 73 and 90 \| |
| --- | --- | --- | --- | --- | --- | --- | --- | --- | --- | --- | --- | --- | --- | --- | --- | --- | --- | --- | --- | --- | --- | --- | --- | --- | --- | --- | --- | --- | --- | --- | --- | --- | --- | --- | --- | --- | --- | --- | --- | --- | --- | --- | --- | --- | --- | --- | --- | --- | --- | --- | --- | --- | --- | --- | --- | --- | --- | --- | --- | --- | --- | --- | --- | --- | --- | --- | --- | --- | --- | --- | --- | --- | --- | --- | --- | --- | --- | --- | --- | --- | --- | --- | --- | --- | --- | --- | --- | --- | --- | --- | --- | --- | --- | --- | --- | --- | --- | --- | --- | --- | --- | --- | --- | --- | --- | --- | --- | --- | --- | --- | --- | --- | --- | --- | --- | --- | --- | --- | --- | --- | --- | --- | --- | --- | --- | --- | --- | --- | --- | --- | --- | --- | --- | --- | --- | --- | --- | --- | --- | --- | --- | --- | --- | --- | --- | --- | --- | --- | --- | --- | --- | --- | --- | --- | --- | --- | --- | --- | --- | --- | --- | --- | --- | --- | --- | --- | --- | --- | --- | --- | --- | --- | --- | --- | --- | --- | --- | --- | --- | --- | --- | --- |

# **EMBASE (OvidSP)**

| 1 | exp self care/ |
| --- | --- |
| 2 | self car*.ti,ab,kw. |
| 3 | self help.ti,ab,kw. |
| 4 | self guided.ti,ab,kw. |
| 5 | self directed.ti,ab,kw. |
| 6 | self manag*.ti,ab,kw. |
| 7 | (patient* adj3 directed).ti,ab,kw. |
| 8 | (patient* adj3 guided).ti,ab,kw. |
| 9 | self improvement.ti,ab,kw. |
| 10 | or/1-9 |
| 11 | exp chronic disease/ |
| 12 | (chronic* adj3 (condition* or disease* or disorder* or illness*)).ti,ab,kw. |
| 13 | (long term adj3 (condition* or disease* or disorder* or illness*)).ti,ab,kw. |
| 14 | (longterm adj3 (condition* or disease* or disorder* or illness*)).ti,ab,kw. |
| 15 | (long standing adj3 (condition* or disease* or disorder* or illness*)).ti,ab,kw. |
| 16 | (longstanding adj3 (condition* or disease* or disorder* or illness*)).ti,ab,kw. |
| 17 | (degenerative adj3 (condition* or disease* or disorder* or illness*)).ti,ab,kw. |
| 18 | (end stage adj3 (condition* or disease* or disorder* or illness*)).ti,ab,kw. |
| 19 | (persistent adj3 (condition* or disease* or disorder* or illness*)).ti,ab,kw. |
| 20 | LTC*.ti,ab,kw. |
| 21 | chronic.hw. |
| 22 | exp epilepsy/ |
| 23 | exp diabetes mellitus/ |
| 24 | exp asthma/ |
| 25 | epilep*.ti,ab,kw. |
| 26 | diabet*.ti,ab,kw. |
| 27 | asthma*.ti,ab,kw. |
| 28 | exp inflammatory bowel disease/ |
| 29 | inflammatory bowel.ti,ab,kw. |
| 30 | IBD.ti,ab,kw. |
| 31 | ulcerative colitis.ti,ab,kw. |
| 32 | crohn*.ti,ab,kw. |
| 33 | stoma/ or colon stoma/ or ileostoma/ |
| 34 | stoma*1.ti,ab,kw. |
| 35 | enterostom*.ti,ab,kw. |
| 36 | ileostom*.ti,ab,kw. |
| 37 | c?ecostom*.ti,ab,kw. |
| 38 | colostom*.ti,ab,kw. |
| 39 | duodenostom*.ti,ab,kw. |
| 40 | jejunostom*.ti,ab,kw. |
| 41 | or/11-40 |
| 42 | young adult/ |
| 43 | adolescent/ |
| 44 | juvenile/ |
| 45 | (early adj3 adult*).ti,ab,kw. |
| 46 | (emerg* adj3 adult*).ti,ab,kw. |
| 47 | adolescen*.ti,ab,kw. |
| 48 | juvenile*.ti,ab,kw. |
| 49 | teen*.ti,ab,kw. |
| 50 | youth*1.ti,ab,kw. |
| 51 | (young adj3 (adult* or person or people or wom#n or m#n)).ti,ab,kw. |
| 52 | or/42-51 |
| 53 | random*.tw. |
| 54 | factorial*.tw. |
| 55 | crossover*.tw. |
| 56 | cross-over*.tw. |
| 57 | placebo*.tw. |
| 58 | (doubl* adj blind*).tw. |
| 59 | (singl* adj blind*).tw. |
| 60 | assign*.tw. |
| 61 | allocat*.tw. |
| 62 | volunteer*.tw. |
| 63 | crossover procedure/ |
| 64 | double blind procedure/ |
| 65 | randomized controlled trial/ |
| 66 | single blind procedure/ |
| 67 | control group/ |
| 68 | time series analysis/ |
| 69 | cohort analysis/ |
| 70 | control*.ti,ab,kw. |
| 71 | exp controlled study/ |
| 72 | (before adj2 after).ti,ab,kw. |
| 73 | (pre adj2 post).ti,ab,kw. |
| 74 | (pretest adj2 posttest).ti,ab,kw. |
| 75 | (pre test adj2 post test).ti,ab,kw. |
| 76 | time series.ti,ab,kw. |
| 77 | cohort.ti,ab,kw. |
| 78 | or/53-77 |
| 79 | 10 and 41 and 52 and 78 |
| 80 | mental health/ |
| 81 | psychological well-being/ |
| 82 | distress syndrome/ |
| 83 | exp physiological stress/ |
| 84 | depression/ or adolescent depression/ |
| 85 | exp anxiety/ |
| 86 | depress*.ti,ab,kw. |
| 87 | distress*.ti,ab,kw. |
| 88 | stress*.ti,ab,kw. |
| 89 | (anxiet* or anxious*).ti,ab,kw. |
| 90 | low mood.ti,ab,kw. |
| 91 | (mental adj2 health).ti,ab,kw. |
| 92 | exp wellbeing/ |
| 93 | (well being or wellbeing).ti,ab,kw. |
| 94 | "quality of life"/ |
| 95 | (quality adj2 life).ti,ab,kw. |
| 96 | (QoL or HRQoL).ti,ab,kw. |
| 97 | life satisfaction.ti,ab,kw. |
| 98 | or/80-97 |
| 99 | 79 and 98 |
| 100 | exp animal/ not human/ |
| 101 | 99 not 100 |
| 102 | limit 101 to embase |

# **HMIC (OvidSP)**

| 1 | exp self care/ | |
| --- | --- | --- |
| 2 | self management/ | |
| 3 | self medication/ | |
| 4 | self car*.ti,ab. | |
| 5 | self help.ti,ab. | |
| 6 | self guided.ti,ab. | |
| 7 | self directed.ti,ab. | |
| 8 | self manag*.ti,ab. | |
| 9 | (patient* adj3 directed).ti,ab. | |
| 10 | (patient* adj3 guided).ti,ab. | |
| 11 | self improvement.ti,ab. | |
| 12 | or/1-11 | |
| 13 | chronic disease/ | |
| 14 | (chronic* adj3 (condition* or disease* or disorder* or illness*)).ti,ab. | |
| 15 | (long term adj3 (condition* or disease* or disorder* or illness*)).ti,ab. | |
| 16 | (longterm adj3 (condition* or disease* or disorder* or illness*)).ti,ab. | |
| 17 | (long standing adj3 (condition* or disease* or disorder* or illness*)).ti,ab. | |
| 18 | (longstanding adj3 (condition* or disease* or disorder* or illness*)).ti,ab. | |
| 19 | (degenerative adj3 (condition* or disease* or disorder* or illness*)).ti,ab. | |
| 20 | (end stage adj3 (condition* or disease* or disorder* or illness*)).ti,ab. | |
| 21 | (persistent adj3 (condition* or disease* or disorder* or illness*)).ti,ab. | |
| 22 | LTC*.ti,ab. | |
| 23 | chronic.hw. | |
| 24 | epilepsy/ | |
| 25 | diabetes/ | |
| 26 | exp asthma/ | |
| 27 | epilep*.ti,ab. | |
| 28 | diabet*.ti,ab. | |
| 29 | asthma*.ti,ab. | |
| 30 | exp intestinal diseases/ | |
| 31 | inflammatory bowel.ti,ab. | |
| 32 | IBD.ti,ab. | |
| 33 | ulcerative colitis.ti,ab. | |
| 34 | crohn*.ti,ab. | |
| 35 | stoma care/ or stoma therapy/ | |
| 36 | stoma*1.ti,ab. | |
| 37 | enterostom*.ti,ab. | |
| 38 | ileostom*.ti,ab. | |
| 39 | c?ecostom*.ti,ab. | |
| 40 | colostom*.ti,ab. | |
| 41 | duodenostom*.ti,ab. | |
| 42 | jejunostom*.ti,ab. | |
| 43 | or/13-42 | |
| 44 | young adults/ | |
| 45 | (young adj3 (adult* or person or people or wom#n or m#n)).ti,ab. | |
| 46 | (early adj3 adult*).ti,ab. | |
| 47 | (emerg* adj3 adult*).ti,ab. | |
| 48 | adolescen*.ti,ab. | |
| 49 | juvenile*.ti,ab. | |
| 50 | teen*.ti,ab. | |
| 51 | youth*1.ti,ab. | |
| 52 | or/44-51 | |
| 53 | 12 and 43 and 52 | |
|  | |  |

# **EBSCO databases**

The following table is an explanation of the symbols used in the EBSCO search strategies below.

MH indicates an index term (CINAHLPlus).

DE indicates an index term (PsycINFO).

+ following term before an index term indicates that all subheadings were selected (CINAHLPlus).

TI indicates a search for a term in the title.

AB indicates a search for a term in the abstract.

KW indicates a search for a term in keywords.

* at the end of a term indicates that this term has been truncated.

*Wn* indicates a search for two terms where they appear with *n* words between them in the given order.

N*n* indicates a search for two terms where they appear with *n* words between them in any order.

# **CINHALPlus (EBSCO)**

| S1 | (MH "Self Care") |
| --- | --- |
| S2 | (MH "Self-Management") |
| S3 | (MH "Self Medication") |
| S4 | TI self W0 car* OR AB self W0 car* |
| S5 | TI "self help" OR AB "self help" |
| S6 | TI "self guided" OR AB "self guided" |
| S7 | TI "self directed" OR AB "self directed" |
| S8 | TI self W0 manag* OR AB self W0 manag* |
| S9 | TI patient* N2 directed OR AB patient* N2 directed |
| S10 | TI patient* N2 guided OR AB patient* N2 guided |
| S11 | TI "self improvement" OR AB "self improvement" |
| S12 | S1 OR S2 OR S3 OR S4 OR S5 OR S6 OR S7 OR S8 OR S9 OR S10 OR S11 |
| S13 | (MH "Chronic Disease+") |
| S14 | MW chronic |
| S15 | TI ( (chronic* N2 (condition* or disease* or disorder* or illness*)) ) OR AB ( (chronic* N2 (condition* or disease* or disorder* or illness*)) ) |
| S16 | TI ( ("long term" N2 (condition* or disease* or disorder* or illness*)) ) OR AB ( ("long term" N2 (condition* or disease* or disorder* or illness*)) ) |
| S17 | TI ( (longterm N2 (condition* or disease* or disorder* or illness*)) ) OR AB ( (longterm N2 (condition* or disease* or disorder* or illness*)) ) |
| S18 | TI ( ("long standing" N2 (condition* or disease* or disorder* or illness*)) ) OR AB ( ("long standing" N2 (condition* or disease* or disorder* or illness*)) ) |
| S19 | TI ( (longstanding N2 (condition* or disease* or disorder* or illness*)) ) OR AB ( (longstanding N2 (condition* or disease* or disorder* or illness*)) ) |
| S20 | TI ( ("end stage" N2 (condition* or disease* or disorder* or illness*)) ) OR AB ( ("end stage" N2 (condition* or disease* or disorder* or illness*)) ) |
| S21 | TI ( (persistent N2 (condition* or disease* or disorder* or illness*)) ) OR AB ( (persistent N2 (condition* or disease* or disorder* or illness*)) ) |
| S22 | TI LTC* OR AB LTC* |
| S23 | (MH "Inflammatory Bowel Diseases+") |
| S24 | TI "inflammatory bowel" OR AB "inflammatory bowel" |
| S25 | TI IBD OR AB IBD |
| S26 | TI "ulcerative colitis" OR AB "ulcerative colitis" |
| S27 | TI crohn* OR AB crohn* |
| S28 | TI ( stoma OR stomas ) OR AB ( stoma OR stomas ) |
| S29 | (MH "Ostomy Care") |
| S30 | TI enterostom* OR AB enterostom* |
| S31 | TI ileostom* OR AB ileostom* |
| S32 | TI ( cecostom* OR caecostom* ) OR AB ( cecostom* OR caecostom* ) |
| S33 | TI colostom* OR AB colostom* |
| S34 | TI duodenostom* OR AB duodenostom* |
| S35 | TI jejunostom* OR AB jejunostom* |
| S36 | (MH "Epilepsy+") |
| S37 | (MH "Diabetes Mellitus+") |
| S38 | (MH "Asthma+") |
| S39 | TI epilep* OR AB epilep* |
| S40 | TI diabet* OR AB diabet* |
| S41 | TI asthma* OR AB asthma* |
| S42 | S13 OR S14 OR S15 OR S16 OR S17 OR S18 OR S19 OR S20 OR S21 OR S22 OR S23 OR S24 OR S25 OR S26 OR S27 OR S28 OR S29 OR S30 OR S31 OR S32 OR S33 OR S34 OR S35 OR S36 OR S37 OR S38 OR S39 OR S40 OR S41 |
| S43 | TI ( youth or youths ) OR AB ( youth or youths ) |
| S44 | TI teen* OR AB teen* |
| S45 | TI juvenile* OR AB juvenile* |
| S46 | TI adolescen* OR AB adolescen* |
| S47 | TI emerg* N2 adult* OR AB emerg* N2 adult* |
| S48 | TI early N2 adult* OR AB early N2 adult* |
| S49 | TI ( (young N2 (adult* or person or people or woman or women or man or men)) ) OR AB ( (young N2 (adult* or person or people or woman or women or man or men)) ) |
| S50 | (MH "Young Adult") |
| S51 | S50 OR S49 OR S48 OR S47 OR S46 OR S45 OR S44 OR S43 |
| S52 | TI control* OR AB control* |
| S53 | TI "pre test" N1 "post test" OR AB "pre test" N1 "post test" |
| S54 | TI pretest N1 posttest OR AB pretest N1 posttest |
| S55 | TI pre N1 post OR AB pre N1 post |
| S56 | TI before N1 after OR AB before N1 after |
| S57 | (MH "Clinical Trials+") |
| S58 | TI random* OR AB random* |
| S59 | TI crossover* OR AB crossover* |
| S60 | TI cross W0 over* OR AB cross W0 over* |
| S61 | TI placebo* OR AB placebo* |
| S62 | TI doubl* W0 blind* OR AB doubl* W0 blind* |
| S63 | TI singl* W0 blind* OR AB singl* W0 blind* |
| S64 | TI assign* OR AB assign* |
| S65 | TI allocat* OR AB allocat* |
| S66 | TI volunteer* OR AB volunteer* |
| S67 | TI ( "time series" OR cohort ) OR ( AB "time series" OR cohort) |
| S68 | S52 OR S53 OR S54 OR S55 OR S56 OR S57 OR S58 OR S59 OR S60 OR S61 OR S62 OR S63 OR S64 OR S65 OR S66 OR S67 |
| S69 | TI ( QoL or HRQoL ) OR AB ( QoL or HRQoL ) |
| S70 | TI quality N1 life OR AB quality N1 life |
| S71 | TI psycho* OR AB psycho* |
| S72 | TI mental N1 health OR AB mental N1 health |
| S73 | TI ( anxiet* OR anxious* ) OR AB ( anxiet* OR anxious* ) |
| S74 | (MH "Anxiety") |
| S75 | (MH "Psychological Well-Being") |
| S76 | (MH "Quality of Life") |
| S77 | TI "low mood" OR AB "low mood" |
| S78 | TI distress* OR AB distress* |
| S79 | TI depress* OR AB depress* |
| S80 | (MH "Depression") |
| S81 | (MH "Psychological Distress") |
| S82 | (MH "Mental Health") |
| S83 | (MH "Stress+") |
| S84 | TI stress* OR AB stress* |
| S85 | TI wellbeing OR AB wellbeing |
| S86 | TI "well being" OR AB "well being" |
| S87 | TI "life satisfaction" OR AB "life satisfaction" |
| S88 | S70 OR S71 OR S72 OR S73 OR S74 OR S75 OR S76 OR S77 OR S78 OR S79 OR S80 OR S81 OR S82 OR S83 OR S84 OR S85 OR S86 OR S87 |
| S89 | S12 AND S42 AND S51 AND S68 AND S88 |

# **PsycINFO (EBSCO)**

| S1 | DE "Self-Care" OR DE "Self-Management" OR DE "Self-Instructional Training" |
| --- | --- |
| S2 | DE "Self-Medication" |
| S3 | TI self W0 car* OR AB self W0 car* OR KW self W0 car* |
| S4 | TI "self help" OR AB "self help" OR KW "self help" |
| S5 | TI "self guided" OR AB "self guided" OR KW "self guided" |
| S6 | TI "self directed" OR AB "self directed" OR KW "self directed" |
| S7 | TI self W0 manag* OR AB self W0 manag* OR KW self W0 manag* |
| S8 | TI patient* N2 directed OR AB patient* N2 directed OR KW patient* N2 directed |
| S9 | TI patient* N2 guided OR AB patient* N2 guided OR KW patient* N2 guided |
| S10 | TI "self improvement" OR AB "self improvement" OR KW "self improvement" |
| S11 | S1 OR S2 OR S3 OR S4 OR S5 OR S6 OR S7 OR S8 OR S9 OR S10 |
| S12 | DE "Chronic Illness" OR DE "Chronic Fatigue Syndrome" OR DE "Chronic Mental Illness" OR DE "Chronic Pain" |
| S13 | MJ chronic |
| S14 | TI LTC* OR AB LTC* |
| S15 | TI ( (persistent N2 (condition* or disease* or disorder* or illness*)) ) OR AB ( (persistent N2 (condition* or disease* or disorder* or illness*)) ) |
| S16 | TI ( ("end stage" N2 (condition* or disease* or disorder* or illness*)) ) OR AB ( ("end stage" N2 (condition* or disease* or disorder* or illness*)) ) |
| S17 | TI ( (longstanding N2 (condition* or disease* or disorder* or illness*)) ) OR AB ( (longstanding N2 (condition* or disease* or disorder* or illness*)) ) |
| S18 | TI ( ("long standing" N2 (condition* or disease* or disorder* or illness*)) ) OR AB ( ("long standing" N2 (condition* or disease* or disorder* or illness*)) ) |
| S19 | TI ( (longterm N2 (condition* or disease* or disorder* or illness*)) ) OR AB ( (longterm N2 (condition* or disease* or disorder* or illness*)) ) |
| S20 | TI ( ("long term" N2 (condition* or disease* or disorder* or illness*)) ) OR AB ( ("long term" N2 (condition* or disease* or disorder* or illness*)) ) |
| S21 | TI ( (chronic* N2 (condition* or disease* or disorder* or illness*)) ) OR AB ( (chronic* N2 (condition* or disease* or disorder* or illness*)) ) |
| S22 | TI "inflammatory bowel" OR AB "inflammatory bowel" |
| S23 | TI IBD OR AB IBD |
| S24 | TI "ulcerative colitis" OR AB "ulcerative colitis" |
| S25 | TI crohn* OR AB crohn* |
| S26 | TI ( stoma OR stomas ) OR AB ( stoma OR stomas ) |
| S27 | TI enterostom* OR AB enterostom* |
| S28 | TI ileostom* OR AB ileostom* |
| S29 | TI caecostom* OR AB caecostom* |
| S30 | TI cecostom* OR AB cecostom* |
| S31 | TI colostom* OR AB colostom* |
| S32 | TI duodenostom* OR AB duodenostom* |
| S33 | TI jejunostom* OR AB jejunostom* |
| S34 | DE "Epilepsy" OR DE "Epileptic Seizures" OR DE "Experimental Epilepsy" OR DE "Lennox Gastaut Syndrome" |
| S35 | DE "Diabetes" OR DE "Diabetes Mellitus" OR DE "Gestational Diabetes" OR DE "Type 2 Diabetes" OR DE "Type 2 Diabetes" |
| S36 | DE "Asthma" |
| S37 | TI epilep* OR AB epilep* |
| S38 | TI diabet* OR AB diabet* |
| S39 | TI asthma* OR AB asthma* |
| S40 | S12 OR S13 OR S14 OR S15 OR S16 OR S17 OR S18 OR S19 OR S20 OR S21 OR S22 OR S23 OR S24 OR S25 OR S26 OR S27 OR S28 OR S29 OR S30 OR S31 OR S32 OR S33 OR S34 OR S35 OR S36 OR S37 OR S38 OR S39 |
| S41 | TI ( youth or youths ) OR AB ( youth or youths ) |
| S42 | TI emerg* N2 adult* OR AB emerg* N2 adult* |
| S43 | TI teen* OR AB teen* |
| S44 | TI juvenile* OR AB juvenile* |
| S45 | TI adolescen* OR AB adolescen* |
| S46 | TI early N2 adult* OR AB early N2 adult* |
| S47 | TI ( young N2 (adult* or person or people or woman or women or man or men) ) OR AB ( young N2 (adult* or person or people or woman or women or man or men) ) |
| S48 | DE "Emerging Adulthood" |
| S49 | S41 OR S42 OR S43 OR S44 OR S45 OR S46 OR S47 OR S48 |
| S50 | DE "Randomized Controlled Trials" OR DE "Clinical Trials" OR DE "Randomized Clinical Trials" |
| S51 | TI volunteer* OR AB volunteer* |
| S52 | TI allocat* OR AB allocat* |
| S53 | TI assign* OR AB assign* |
| S54 | TI singl* W0 blind* OR AB singl* W0 blind* |
| S55 | TI doubl* W0 blind* OR AB doubl* W0 blind* |
| S56 | TI placebo* OR AB placebo* |
| S57 | TI cross W0 over* OR AB cross W0 over* |
| S58 | TI crossover* OR AB crossover* |
| S59 | TI factorial* OR AB factorial* |
| S60 | TI random* OR AB random* |
| S61 | TI before N1 after OR AB before N1 after |
| S62 | TI pre N1 post OR AB pre N1 post |
| S63 | TI pretest N1 posttest OR AB pretest N1 posttest |
| S64 | TI "pre test" N1 "post test" OR AB "pre test" N1 "post test" |
| S65 | TI control* OR AB control* |
| S66 | DE "Time Series" |
| S67 | DE "Treatment Effectiveness Evaluation" |
| S68 | DE "Cohort Analysis" |
| S69 | TI cohort OR AB cohort |
| S70 | S50 OR S51 OR S52 OR S53 OR S54 OR S55 OR S56 OR S57 OR S58 OR S59 OR S60 OR S61 OR S62 OR S63 OR S64 OR S65 OR S66 OR S67 OR S68 OR S69 |
| S71 | S11 AND S40 AND S49 AND S70 |

# **Cochrane Library**

#1 MeSH descriptor: [Self Care] this term only

#2 MeSH descriptor: [Self-Management] this term only

#3 MeSH descriptor: [Self Medication] this term only

#4 self NEXT car*:ti,ab,kw

#5 "self help":ti,ab,kw

#6 "self guided":ti,ab,kw

#7 "self directed":ti,ab,kw

#8 self NEXT manag*:ti,ab,kw

#9 patient* near/3 directed:ti,ab,kw

#10 patient* near/3 guided:ti,ab,kw

#11 "self improvement":ti,ab,kw

#12 {OR #1-#11}

#13 MeSH descriptor: [Chronic Disease] explode all trees

#14 (chronic* NEAR/3 (condition* or disease* or disorder* or illness*)):ti,ab,kw

#15 ("long term" NEAR/3 (condition* or disease* or disorder* or illness*)):ti,ab,kw

#16 (longterm NEAR/3 (condition* or disease* or disorder* or illness*)):ti,ab,kw

#17 ("long standing" NEAR/3 (condition* or disease* or disorder* or illness*)):ti,ab,kw

#18 (longstanding NEAR/3 (condition* or disease* or disorder* or illness*)):ti,ab,kw

#19 (degenerative NEAR/3 (condition* or disease* or disorder* or illness*)):ti,ab,kw

#20 ("end stage" NEAR/3 (condition* or disease* or disorder* or illness*)):ti,ab,kw

#21 (persistent NEAR/3 (condition* or disease* or disorder* or illness*)):ti,ab,kw

#22 LTC*:ti,ab,kw

#23 MeSH descriptor: [Epilepsy] explode all trees

#24 MeSH descriptor: [Diabetes Mellitus] explode all trees

#25 MeSH descriptor: [Asthma] explode all trees

#26 epilep*:ti,ab,kw

#27 diabet*:ti,ab,kw

#28 asthma*:ti,ab,kw

#29 MeSH descriptor: [Inflammatory Bowel Diseases] explode all trees

#30 "inflammatory bowel":ti,ab,kw

#31 IBD:ti,ab,kw

#32 "ulcerative colitis":ti,ab,kw

#33 crohn*:ti,ab,kw

#34 MeSH descriptor: [Enterostomy] explode all trees

#35 (stoma OR stomas):ti,ab,kw

#36 enterostom*:ti,ab,kw

#37 ileostom*:ti,ab,kw

#38 (cecostom* OR caecostom*):ti,ab,kw

#39 colostom*:ti,ab,kw

#40 duodenostom*:ti,ab,kw

#41 jejunostom*:ti,ab,kw

#42 {OR #13-#41}

#43 MeSH descriptor: [Young Adult] this term only

#44 MeSH descriptor: [Adolescent] this term only

#45 young NEAR/3 (adult* OR person OR people OR woman OR women OR men OR man):ti,ab,kw

#46 early NEAR/3 adult*:ti,ab,kw

#47 emerg* NEAR/3 adult*:ti,ab,kw

#48 adolescen*:ti,ab,kw

#49 juvenile*:ti,ab,kw

#50 teen*:ti,ab,kw

#51 (youth OR youths):ti,ab,kw

#52 {OR #43-#51}

#53 #12 AND #42 AND #52

#54 MeSH descriptor: [Mental Health] this term only

#55 MeSH descriptor: [Psychological Distress] this term only

#56 MeSH descriptor: [Stress, Psychological] explode all trees

#57 MeSH descriptor: [Depression] this term only

#58 MeSH descriptor: [Anxiety] this term only

#59 depress*:ti,ab,kw

#60 distress*:ti,ab,kw

#61 stress*:ti,ab,kw

#62 "low mood":ti,ab,kw

#63 (anxiet* OR anxious*):ti,ab,kw

#64 mental near/2 health:ti,ab,kw

#65 "life satisfaction":ti,ab,kw

#66 MeSH descriptor: [Quality of Life] this term only

#67 quality near/2 life:ti,ab,kw

#68 (QoL OR HRQoL):ti,ab,kw

#69 ("well being" OR wellbeing):ti,ab,kw

#70 {OR #54-#69}

#71 #53 AND #70
