# Supplementary material for: Self‐directed self‐management interventions to prevent or address distress in young people with long‐term physical conditions: A rapid review
Source: Health Expect. 2023 Aug 21;26(6):2164–90. doi: 10.1111/hex.13845 (PMC10632640; doi:10.1111/hex.13845)
Supplement: Supplementary file 2 — Supporting information. [file HEX-26--s003.docx]

**Supplementary file 2:** Characteristics of interventions (detailed). **Abbreviations:** AZA/MES - azathioprine/mesalamine; BCT - Behaviour change technique; CBT – Cognitive behavioural therapy; CF - Cystic fibrosis; HRQoL – Health related quality of life; IBD – inflammatory bowel disease; ITT – intention to treat; mHealth apps - mobile health applications; NR – not reported; PABA - Perceptions and Practicalities Approach; QoL – Quality of Life; SM – self-management; SMS – short message service; T1DM – Type I diabetes mellitus; T2DM – Type II diabetes mellitus; TMD - Temporomandibular disorder; YLWH - Young people living with HIV. If author is **emboldened** then the intervention was significantly more beneficial regarding outcomes of interest compared to control.

| Study ID year | Intervention | Why  Study aim, theories/frameworks informing the intervention | How  Mode of delivery and whether provided individually or in a group | What materials  Physical or informational materials used, incl. those provided to participants or used in intervention delivery or in training of intervention providers | What procedures  Procedures, activities, and/or processes used in the intervention, incl. enabling or support activities | Who provided  Intervention provider incl. expertise, background and training given | Where  Type(s) of location(s) where the intervention occurred | When and how much  Nos. times the intervention was delivered and over what period incl. number of sessions, their schedule, and their duration, intensity or dose | Tailoring  If the intervention was planned to be personalised, titrated or adapted | How well planned  If adherence or fidelity assessed, describe how and by whom, and describe any strategies were used to maintain or improve fidelity | How well actual  If adherence or fidelity was assessed, describe the extent to which the intervention was delivered as planned |
| --- | --- | --- | --- | --- | --- | --- | --- | --- | --- | --- | --- |
| **Ayar**  **2021^24^** | “Youth diabetes” - web-based diabetes education + standard medical care | To examine whether web-based diabetes education was effective in improving metabolic control, self-efficacy for diabetes self-management, and QoL in adolescents with T1DM. | Web-based for individuals, but with an area where patients able to chat to each other | Multimedia learning tools e.g., tutorials using PowerPoint about diabetes management, quizzes, and blogs.  New topics (e.g., definition of the disease, nutritional aspects, carbohydrate counting, management of acute and chronic complications, insulin regimen, physical exercise, and self-management) and relevant blog texts were added to the site every week  Topics addressed on the blog site were selected from the literature and included e.g. “What is diabetes in your opinion?”; “Is exercise important in the management of type 1 diabetes?”; “Let’s share our experiences: how do you know when you have high blood glucose?”; “How do you know when you have low blood glucose?”  The appropriateness and sufficiency of educational topics uploaded to web site were examined by 11 experts (including paediatric endocrinologists, professors working on paediatric nursing, diabetes clinic nurses, and the head of the diabetes association in Turkey). Content validity index regarding educational content was 0.96, suggesting high agreement among experts. | Adolescents were introduced to the "youth diabetes" website and told to use as part of their polyclinic appointment. They could log in at any time.  **Member panel:** user homepage with username and password (provided by researcher) to log on. The website included a series of learning objectives which used multimedia learning tools. There was also a blog where adolescents could chat and share experiences. [asked to comment and share experiences on issues suggested by researchers].  Adolescents were encouraged to log on at least twice a week, and a reminder message was sent when new materials were added to the website (no reply expected).  **Administrator panel:** elements required for performance, organization, and implementation of operations. It had menu elements, user operations, and unanswered survey forms as well as the content display. Also used to add and delete users and update the website.  The researcher was notified when someone posted a comment on the blog. Comments were published on the website under the supervision of the researcher. | Researchers administered the intervention: determining and updating information on the website; also suggested topics for discussion on blogs | NR | Delivered over a 6-month period. Participants encouraged to log in ≥2 time a week to update blogs.  A reminder message was sent each time new material was added to the website | None | n/a | n/a |
| **Balato**  **2013^25^** | Text messages | To evaluate use of Text Messages in improving treatment adherence and patient outcomes such as QoL, disease severity, patient-perceived disease severity and the patient–physician relationship. | Mobile text messages, individual | Text messages created using simple language, considering frequently asked questions about psoriatic drugs (e.g. adverse effects, administration) and general advice to take care of overall health. | Text messages with either an educational tool or reminder were sent daily | Investigators were responsible for sending text messages | NR | 1 text message daily for 12 weeks, in the same randomly selected order (reminders 3 x weekly, education tools 4 x weekly) | None | n/a | n/a |
| Bell  2021^26^ | Narrative care messages | To determine if narratives would provide a better tool to improve disease management for adolescents with T1DM. | Online, individual | Messages presented online through an ipad.  Narratives focus on increased blood sugar testing and obtaining optimal glycaemic control, encouraging open communication with healthcare providers about issues in managing T1DM, and social support and overcoming perceptions of social stigma about disease management.  Narratives were based entirely on the lived experiences of young adults with T1DM. Each narrative started with the diagnosis of T1DM, the new reality that the diagnosis brought, growing up with the disease, and the barriers each character faced, including erratic blood sugar levels, detrimental health impacts, negative outcome expectations, and general lifestyle activities that come with being a teenager. The issues outlined in the narratives were all in line with the literature around issues facing adolescents with T1DM and experiences seen by healthcare experts. Stories concluded with a change in health behaviour, resulting from conflict, when they were younger and justification for why that behaviour has been successful for them now. (nb: messages used in study in Figure 1 & supplement).  Text-based narratives averaged 450 words each and were written in the first person. They did not include any graphics; however, some key elements were bolded to draw emphasis to the key messages.  Participants were given a $5 gift card and a chance to win one of three $50 gift cards (if recruited via clinic or online support sites) or standard panel incentive for participation (if recruited via Qualtrics). | If recruited via clinic, participants took part in the study through an iPad. If recruited online a direct message was sent to the page administrators to disperse study information to parents. Once verified, a unique link to the study was sent to the parent.  After completing baseline questionnaires, participants saw 3 consecutive messages concerning their condition. To ensure there was enough time to fully read each, participants were not allowed to advance to the next screen until at least 30 seconds had passed, but they could take as much time as needed. Upon viewing all three messages, participants were immediately asked to recall three things about the messages to gauge message receptivity.  Following cued recall, all participants answered questions regarding their emotional reaction and the perceived effectiveness of the message. After message evaluation, they answered questions about self-efficacy, followed by negative outcome expectations, positive outcome expectations, and stress and burnout perceptions. The experiment ended with participants answering questions about their behavioural intentions regarding T1DM management. | n/a | NR | One off, immediate response | None | n/a | n/a |
| **Chapman**  **2020^27^** | Tailored digital intervention to change adherence-related beliefs and barriers | The study aimed to develop and assess a tailored digital intervention (algorithm) to support adherence to maintenance treatment for patients with IBD.  The intervention employed a Perceptions and Practicalities Approach (PABA) and was informed by Horne’s Necessity-Concerns Framework and utilised several Behaviour Change Techniques. | Online-platform, individual | The intervention incorporated personalised messages to address beliefs about IBD and maintenance treatment and to provide advice on the practical difficulties with taking regular medication. It consisted of three-stages corresponding to the three-components of the PABA model:   1. Necessity - provide a rationale for the need of medication so patients perceive a ‘common-sense’ fit between IBD and treatment; 2. Concerns - elicit and address concerns about IBD medication such as potential adverse effects; 3. Practical barriers – address practical issues/barriers to taking medication in daily life.   In addition, an IBD Library was provide that contained general resources about living with IBD to complement the adherence support.  After the study started, dropout rate was higher than expected, so a prize draw of £150 online gift voucher was introduced for participants who completed all follow-ups. | Following randomisation participants completed baseline assessments and undertook a 3-month online intervention.  Behaviour Change Techniques (BCT) used in each stage:  **Stage 1: Treatment need session** BCT: Credible source - Strategies: Present quotes from IBD experts to explain why treatment is needed during flare-ups and remission BCT: Information about health consequences - Strategies: Provide information re negative health consequences of not taking AZA/MES and positive health consequences of taking them in the short and long-term BCT: Pharmacological support - Strategies: Promote the understanding of why IBD treatment is needed and encourage adherence to AZA/MES  BCT: Pros and cons - Strategies: Address decisional balance and identify reasons for wanting and not wanting to take AZA/MES as prescribed BCT: Social support (unspecified) - Strategies: Advise participants to contact their IBD team to discuss their doubts about AZA/MES **Stage 2: Concerns session** BCT: Pros and cons - Strategies: Present written and pictorial information about the advantages and disadvantages of taking AZA/MES. Present evidence for and against each concern reported at baseline BCT: Problem solving - Strategies: Explore aspects underlying each concern about taking prescribed medication and explore ways to cope BCT: Social support (unspecified) - Strategies: Advise participants to contact their IBD team to discuss self-management of side effects and ways to cope with their concerns about AZA/MES BCT: Framing/reframing - Strategies: Address negative thought processes and suggest adoption of a more realistic way of thinking in order to diminish the concerns about their prescribed medication (cognitive restructuring) BCT: Credible source - Strategies: Provide quotes from IBD experts to present evidence based information for concerns surrounding the prescribed medication  BCT: Information about health consequences - Strategies: Present information about the consequences of not taking their prescribed medication or not doing regular check-ups with their IBD team BCT: Pharmacological support - Strategies: Encourage vaccinations in order to diminish concerns about getting infections while taking azathioprine **Stage 3: Practical issues session** BCT: Problem solving - Strategies: Explore participant’s practical barriers to medication adherence and how to overcome them. Advise to identify the main doubts before a doctor’s appointment and how to overcome difficulties during the appointment to get the best from the consultation BCT: Prompts/cues - Strategies: Define environmental stimulus with the purpose of improving medication adherence BCT: Action planning - Strategies: Prompt planning performance of a particular daily activity at a specific time and linking this with taking their medication BCT: Social support (unspecified) - Strategies: Advise to contact their IBD team to discuss any adjustments to their medical regimen BCT: Self-monitoring of behaviour - Strategies: Explain how a medication diary works and ask the participant to complete it every day to keep track of their medication intake BCT: Restructuring the physical environment - Strategies: Advise to organise and store tablets in a dosette box to help them to take their medication BCT: Habit formation - Strategies: Prompt participants to take their medications as part of their daily routine BCT: Goal setting (behaviour) - Strategies: Set goals to take their medication daily as prescribed BCT: Social support (practical) - Strategies: Recommend the use of Prescription Prepayment Certificates to cope with the medication costs. Arrange help from friends, partner or relatives to remember to take their medication. Recommend the use of apps or programmes to set up reminders to take their medication BCT: Behavioural practice/rehearsal - Strategies: Prompt practice to swallow tablets by using sweets **IBD library session** BCT: Framing/reframing - Strategies: Suggest ways to replace unhelpful thoughts with more realistic thoughts about IBD and medication  BCT: Social support (unspecified) - Strategies: Advise to discuss with IBD team strategies to cope with flare-ups and IBD symptoms linked to medication adherence. Advise on ways of coping with a flare-up and recommend national services that can provide support during these phases BCT: Demonstration of the behaviour - Strategies: Present a video that shows how to use enemas and suppositories. BCT: Pros and cons - Strategies: Explore advantages and disadvantages of treatment for IBD BCT: Information about health consequences - Strategies: Explain how the body is affected by IBD and how the prescribed medication helps the body to achieve and maintain remission BCT: Social support (emotional) - Strategies: Advise on sharing their concerns and worries about IBD and its treatment with friends, family, partner and/or support groups BCT: Action planning - Strategies: Prompt participants to plan how to cope with a flare-up and IBD symptoms including medication-taking (e.g. developing self-management plans) BCT: Focus on past success - Strategies: Advise to describe successful ways they used to cope with stressful feelings caused by IBD and the prescribed medication  Communication strategy was based on CBT and motivational interviewing, to ensure BCTs were delivered using language that would enhance awareness and motivation. | n/a | NR | As and when over 3 months  (at patients’ convenience) | Intervention designed to be tailored: content of the messages was personalised using the Persignia algorithm which tailored content to address specific perceptual and practical barriers identified by a pre-screening tool. | Intervention usages was recorded automatically (noting the time each page of the site was accessed). This information was used to calculate the total time spent accessing the website by each participant, and check when the intervention content was accessed over the follow-up period *i.e.* total number of visits, total time spent, and date of first access. | Intervention used by 73.2% [n = 112] of the Intervention Group. For those logging on, the maximum number of sessions was 5; 54.9% logged on once, with the remaining participants using the intervention on multiple occasions.  Median total time spent on the website was 9.36 mins (range <0.01 s to 73 min).  Participants accessed a median of 22 pages [range 1–124].  Those never logging on did not differ significantly in terms of any baseline characteristic measured (all p >0.05).  Most frequently visited area of the website was the Practical Barriers section (75.9% of participants); The Concerns section was accessed by 56.3%, the Necessity sections by 45.5%, and the IBD library section by 34.8%. |
| Dilorio  2011^28^ | WebEase - theory-based, interactive, internet-based self-management program for people with epilepsy | The study aimed to determine if individuals with epilepsy who participated in WebEase (Web Epilepsy Awareness, Support, and Education) program demonstrated improvements in medication adherence, perceived stress, and sleep quality.  WebEase was created to provide self-management education and support for people with epilepsy. It was based on self-management models and self-determination principles and incorporated concepts and principles from three theoretical perspectives: social cognitive theory, the transtheoretical model of behaviour change, and motivational interviewing. | Interactive, web-based program; individual | WebEase core program comprises of MyLog application and 3 modules: medication management, stress management, and sleep-management.  The Resource component of WebEase supplements the modules. As participants progress through the modules, they can select a variety of learning strategies e.g. reading information, listening to audiofiles of people with epilepsy, or linking to epilepsy sites such as those of the Centers for Disease Control and Prevention and the Epilepsy Foundation. | After logging into WebEase, participants are first required to complete ‘MyLog’ to record information about seizures, medication taking, stress, and sleep quality ratings. Following completion, they can access other components of WebEase, including the modules.  Daily information is entered every time they log onto WebEase. Data are stored in a database and can be viewed by participants in text and graphic format. Information entered in MyLog is also used to provide feedback in the Module session.  As participants work through each module, they assess their current status (i.e., Looking At My Medications), reflect on current behaviours (i.e., Thinking About My Medications), decide whether or not to change behaviour, and create a goal and action plan either to maintain current behaviour or to change behaviour (i.e., Planning The Next Steps). Modules designed to correspond with stages of change i.e. precontemplation, contemplation, preparation, action, and maintenance, as determined by information entered into the introductory portion of each module and from that entered into MyLog.  The five stages differ in types of activities and emphasis given to considering change in behaviours. E.g. the focus of the precontemplation stage is to support positive attitudes and encourage people to think about adopting healthy behaviours; whilst the contemplation stage gets participants to compare benefits and costs of behaviours and to encourage thinking about strategies to support healthy behaviours. The content covered in each module is delivered using MI principles (e.g. after entering the benefits of stress reduction for them, they then receive reflective feedback restating what they said).  Participants were sent weekly reminders to log into the site if they failed to do so immediately after receiving the initial invitation. Those who had logged into the WebEase site were sent weekly reminders to continue working through the modules and exploring the site resources.  In addition, to engage participants in learning about epilepsy, there was a daily poll questions and short quizzes that could be accessed from the homepage.  At the end of 6 weeks, access to the program for participants was ended.  Participants received an Amazon gift card at the end of their participation in the study. | n/a | NR | 6 weeks. Program set so each participant spent 2 weeks in each of the 3 core modules (medication, stress, sleep management) | None | n/a | n/a |
| Hockemeyer  2014^29^ | Self-administered manual-based stress management intervention | To develop and examine the feasibility and effectiveness of a complementary, self-administered, manual-based intervention for asthmatic college students, which incorporated three major treatment components: relaxation training, CBT, and written emotional expression. | Workbook in own time | Workbook consisted of 3 components:   1. audiocassette tape that included a 20-minute deep-breathing relaxation-training exercise - previously found beneficial for individuals with asthma (plus portable cassette player if participant did not have access to one). 2. CBT exercises: included brief (1-2 pages) reading each week to help participants become aware of their thoughts, feelings, and emotions about stressful experiences. Each week’s reading had a corresponding application on which participants were instructed to spend 30 minutes working. The applications focused on recording thoughts and completing cognitive restructuring exercises. Choose these CBT exercises because they could be adapted into a format that participants could easily perform independently without needing to interact with another individual. 3. 20-minute writing exercises were integrated into the treatment workbook to complement the CBT exercises for each of the 4 weeks.   Nb: participants were given course credit (if psychology students) or paid $10 on completion | Following initial session for baseline assessments, participants undertook 4-week book intervention.  Skills-training and writing exercises completed once a week for each of the 4 weeks.  Workbook instructions prompted participants to complete either the CBT or problem-solving skills on the 1^st^ or 2^nd^ day of the week in each of the 4 weeks (eg, Monday or Tuesday). One to 2 days after completing the CBT or problem-solving skills (eg, Wednesday or Thursday), they were instructed to complete the writing exercises.  Writing exercises:   - Week 1: Identify an experience that continues to be the most stressful to you at this point in your life, describing the experience in detail. - Week 2: Write for another 20 minutes about the stressful experience you wrote about last week, especially your deepest feelings associated with the event. - Week 3: Continue to write for 20 minutes about your stressful experience and the various emotions that you felt. This week, you should also think and write about the beliefs you have developed because of the stressful experience. - Week 4: Continue to think and write about your stressful experience and your feelings for the next 20 minutes; this week, you should question or challenge the beliefs you have had surrounding your stressful experience. | n/a | At home or residence | 4-week period: participants completed CBT/ problem-solving skills on the 1^st^ or 2^nd^ day of the week in each of the 4 weeks.  One to 2 days after completing this, they were instructed to complete the writing exercises. | None | Overall compliance measured by counting the number of weeks participants performed all workbook activities.  Also, number of weeks participants completed their writing components. | Significant differences in compliance between participants who received extra credit and those who were paid $10.  90% of the extra-credit participants had perfect workbook compliance, whereas 50% of paid participants had perfect compliance. |
| **Huang**  **2014^30^** | MD2Me - technology based disease management intervention | The study aim was to evaluate a generic, internet- and mobile phone–delivered disease management intervention aimed at improving disease management, self-efficacy, and communication in adolescents with chronic disease (IBD, CF, T1DM - chosen to represent broad disease spectrum).  Intervention based on Bandura's Social Cognition Theory, targeting self-management constructs of disease symptom monitoring, responding to monitoring with appropriate treatments, and actively working with health care providers to manage care. | Website and text messages.  nb: Mobiles and plans were provided to MD2Me subjects who did not have a mobile phone. | Website provided theme-based materials outlining common disease management and communication skills, and lifestyle tips (see example below). Disease-specific case studies were provided to increase usability.  Example tip sheet: addressing the management of pain, anxiety, and stress. Sheet gave background information and then suggests how to reduce stress and anxiety incl. problem-solving skills (learnt about in week 3) i.e identification of source of stress, identification of problem, brainstorm solutions and making a plan. Suggests working with the medical team to devise a management plan, if stress is caused by the condition such as, to change medication, focusing on stress reduction and ways to relax body and mind e.g. writing, letting feelings out, do something you enjoy, focus on present, exercise, practice breathing exercises e.g. deep breathing, yoga or tai chi; and to maintain healthy eating. Other ways suggested including sign posting to support groups or counselling or therapy. The tip sheet also highlights that whilst people may use alcohol, other substances or social isolation to deal with stress, these are of short term benefit and do not work in the long term. | Participants were asked to log in to a secure website weekly for 2 months, where they received theme-based materials. 3-5 tailored SMS messages and queries were sent each week, to ensure that participants received and understood intervention messages.  After 2 months, website access was provided as a disease management and information resource. Weekly reminder SMS messages were also delivered to reinforce previously introduced concepts and skills.  To facilitate patient-initiated communication, participants were given access to an automated SMS algorithm that provided disease management decision support and a health care team communications portal.  SMS could be activated to report health concerns. Depending on level of urgency (ie, nonurgent [to be addressed within 48-72 hours] or urgent [requiring same-day attention]) participant concerns were relayed to the health care team (by page or e-mail) for intervention.  Usual health care communication portals were available to all participants (including controls). | n/a | NR | 1st 2 months: log on to website weekly; and received tailored SMS 3-5 a week | Tailored to specific disease i.e. IBD, CF or T1DM  No modification reported. | Compliance and performance monitored with regards accessing website and SMS curricula and SMS algorithm. | 79% (median) of curriculum receipt was noted across both web and SMS.  Most web visits occurred during the initial 2 months (97% users visited the website over the first 2 months, but only 42% users visited in the latter 6 months).  In contrast, all but 1 user (97%) responded to SMS curricula with 78% compliance.  77% used the SMS algorithm (users were significantly older than those who did not use the SMS algorithm (p=0.01) |
| **Hunt**  **2020^31^** | Self-help CBT book | To determine the effectiveness of a self-help CBT workbook for patients with IBD. | Self-help book, individual | Self-help book modules:   - Module 1 – Workbook introduction, differential diagnosis, psychoeducation about the effect of stress on the intestines, relaxation exercises - Module 2 – Introduces basic cognitive model of stress management. Discusses “catastrophic cognitions” and introduces cognitive restructuring. Asks participants to complete and practice thought records. Participants describe a negative event, list the thoughts caused by the event, list the feelings caused by those thoughts, and then list a potential alternative explanation for the event that is more objective - Module 3 - Uses cognitive restructuring to the context of GI symptoms and IBD. Participants are asked to complete more thought records. - Module 4 - Introduces behavioural experiments. Participants asked to identify a negative belief about their GI symptoms, predict what will happen if that belief is true, test the relevant situation, and compare what happens to the prediction. - Module 5 - Discusses avoidance behaviours and how and why to eliminate them. - Module 6 - Discusses diet, medical treatment options, and ostomy considerations. Concludes with final thoughts and summarises importance of practicing relaxation strategies, objectively looking at negative thoughts, completing behavioural experiments, and eliminating avoidance. | After randomisation participants received an email informing them of their group allocation and including a link to the first module of their assigned book.  At end of each module there was a short, entertaining “quiz”, which allowed assessment of whether participants had read the material all the way through.  Participants were given 6 weeks to work through the material.  Participants were allowed to maintain their access to the workbooks for the duration of the trial, just as a self-help book would remain in the person’s possession after purchase. | n/a | NR | As and when over the 6 weeks | None | n/a | n/a |
| Joseph  2007^32^ | Puff city - multimedia, web-based asthma management program | The study aimed to develop and evaluate a multimedia, web-based asthma management program (Puff City) to specifically target urban high school students.  To motivate behavioural change, tailoring is used to apply the concepts of the transtheoretical model, and the health belief model. The intervention focuses on 3 core behaviours: controller medication adherence, rescue inhaler availability, and smoking cessation/reduction. | Online, individual | Program content - based on recommendations for patient education in national guidelines and other nationally accredited sources.  Focuses on 3 core behaviours: controller medication adherence; rescue inhaler availability; smoking cessation/reduction.  Consists of 4 consecutive educational computer sessions that make use of both normative (“compared with other students”) and ipsative feedback (“compared with your last session”).  Messages are voiced over to accommodate low literacy. | Following randomisation, students were given 180 days baseline to complete the four sessions. | n/a | Students access program using computers at participating schools | 180 days given to complete all 4 sessions | Yes. Participant specific information necessary for tailoring is obtained at baseline and during the four sessions. | Study compliance measured as failed to complete even 1 session; completed all 4 sessions; completed 12-month follow up; completed baseline and 12-months follow up in same season | Treatment students were more likely to complete all four sessions compared with control students.  The proportion of treatment students completing the 12-month follow-up, or completing baseline and follow-up in the same season, did not differ from that of control students. |
| Klee  2018^33^ | Webdia - Patient-Designed Do-It-Yourself Mobile Device App | To evaluate the impact of a multidisciplinary intervention consisting of using Webdia, a patient-designed mHealth app for smartphones, combined with an educational intervention by specialized nurses and regular insulin dose adaptation by diabetologists on metabolic control of T1DM, QoL, and frequency of hypoglycemia in children.  Webdia was developed by a father whose 10-year-old daughter was diagnosed with T1DM, with the aim of improving his daughter’s autonomy and facilitate data exchange within the family. | Smartphone app | Webdia consists of a simple interface making its use possible for children ≥10 years.  Webdia’s main features are   1. Bolus calculator: uses blood glucose readings and amount of ingested carbohydrates, to calculate the insulin dose needed. 2. “Meals” section: comprehensive list of nutrients and their carbohydrate content. Pictures of 18 common meals on a standard plate to permit comparison of the picture with the content of a given plate, to allow estimate of carbohydrate content. 3. “Favourites” section: allows the user to save frequently eaten meals and their correspondent carbohydrate content. 4. “Data” section: allows user to review last entries for blood glucose, ingested carbohydrates and the calculated insulin dose. 5. Automatic data transfer: All data entered by the user is continuously transmitted to a secure server and becomes available to the user and anyone they agree to share their account with, on a secure dedicated website (<http://www.webdia.ch>): this includes consultants/doctors who have a dedicated interface, which allows them to remotely access patient data, provided Pt has granted them access. | An initial tutorial included installation of Webdia onto ≥1 personal mobile devices, setting up the application, an introduction to app functions, and creation of a user account that allowed for remote access to blood glucose data by both the patient and diabetes team.  Participants used Webdia as often as possible for 3 months, during which time they were called after 1 month to ensure there were no technical problems and to answer any questions (specific to study, not the standalone app).  Blood glucose values were reviewed every month by the diabetic consultants and suggestions to adapt treatment were sent to participants via e-mail. E-mail content was standardised and always consisted of a comment on glucose values and, if necessary, a suggestion to adjust the insulin regimen by changing the application’s settings.  Outside the intervention period, participants were prohibited from using Webdia, and the absence of usage was ascertained by the diabetic team. | Initial tutorial given by two different specialised nurses - to ensure similar delivery of information. | NR | Initial tutorial - one off, lasted 45 minutes Webdia - to use as often as possible for 3 months. | None | Participants were considered “non-compliant” if the application was used less than 4 days per week. | 80% participants reported using Webdia every day |
| Lam  2020^34^ | Internet based multimodal pain program | To investigate the treatment effect of an internet-based multimodal pain program on chronic TMD pain and evaluate the feasibility of a larger randomized controlled trial.  The intervention was based on cognitive behaviour therapy and self-management principles and adapts face-to-face therapy to an online platform (software program), designed to be used without guidance. | Online, individual | Intervention consists of 7 modules and offline activities included homework assignments in a paperback workbook:   - Module 1: Introduction. *Info:* introduction to the treatment program and CBT, goal setting; *CBT component:* Values and goals; *Assignment:* reflection on previous strategies of handling TMD pain, assessment of core values. - Module 2: What is face & jaw pain: *Info:* etiology and epidemiology of TMD pain, anatomy of the masticatory system; *CBT component:* Psychoeducation and assessment; *Assignment:* pain drawing, identify jaw functions limited by pain. - Module 3: What affects my pain in the face and jaw? *Info:* modulating and maintaining factors of TMD pain, pain physiology, acute and chronic pain, the link between quality of life and TMD pain; *CBT component:* Psychoeducation and assessment; *Assignment:* 7-day pain diary - Module 4: How can I relieve my pain? *Info:* treatment alternatives including analgesics, relaxation, jaw exercises, different occlusal splints, acupuncture, and massage; *CBT component:* Applied relaxation and skills training; *Assignment:* identify pain modulators from pain diary, practice relaxation, and jaw exercises - Module 5: The relation between stress and pain; *Info:* stress responses, the link between stress and pain; *CBT component:* Functional analysis, problem-solving, relaxation, skills training; *Assignment:* a questionnaire to assess the degree of stress, situational analyses, list desired ways to enhance recuperation, continue relaxation and jaw exercises - Module 6: How can I reduce my stress and pain levels?; *Info:* stress and pain management; breathing exercises, diet, sleep, training, time management, setting boundaries and acceptance, continued relaxation and jaw exercises; *CBT component:* Committed action, relaxation, skills training; *Assignment:* mapping sleep, training and dietary habits in a weekly schedule and do an activity plan aiming to make desired changes regarding these, continued relaxation and jaw exercises - Module 7: Summary and a plan for future action; *Info:* Setbacks and maintenance planning, summary of the program; *CBT component:* Summary and maintenance plan; *Assignment:* Develop a maintenance plan | Those allocated to internet-based program were sent working material by mail and treatment was started with assistance provided by phone. The start-up phone call, guided participants through the functions in the program and they informed about time requirements for the treatment.  Access required 2-factor authentication. Intervention consists of 7 modules and offline activities included homework assignments in a paperback workbook (see previous column re activities associated with each module).  Whilst the intervention was designed to be used without guidance, to support study adherence telephone, email, and asynchronous chat support was provided by a dentist. After finished each module, participants received an individualised follow-up phone call to provide support and feedback. If participants were unreachable at scheduled follow-up calls, a message was sent via the chat function in the program prompting them to get in touch. If no contact was made in the subsequent 2 weeks, the dentist tried to contact the participant by phone. If they were still unreachable, access to the internet-based multimodal pain program was removed, and the participant was withdrawn from the study. A letter was sent to the participant with information about the withdrawal and a prompt to seek treatment from a dentist.  nb: screenshots given which include jaw exercise instructional video | To support study adherence: dentist who had received 2-day training on internet-based CBT provided support (email, phone, chat) | NR | The intended treatment duration was 7 weeks: 1 module/week; 40 minutes/module online plus time for homework assignments. | None | n/a | Of the 20 participants randomized to the internet-based multimodal pain program, 14 started treatment and 8 completed all 7 modules of the program |
| Linden  2018^35^ | Person-centred, web-based support programme | To determine the effectiveness of a person-centred, web-based support intervention to be used during pregnancy and in early motherhood by women with T1DM. The intervention focused on strengthening autonomy and personal capacity, thereby optimising well-being and self-efficacy of diabetes management. Further, the study aimed to explore the use of the web-based support.  The intervention was based on person-centred care, and designed to assist in decision-making, to support self-care and to facilitate peer contact. | Online, mainly individual (some peer support) | Three components:   1. Evidence-based information on 3 topics: being pregnant, labour and childbirth, and life as a new mother with diabetes. 2. A self-care diary for self-reported monitoring of blood glucose, insulin doses, diet, activities, and daily mood measures that could be viewed and evaluated in tables and diagrams. This was designed as an alternative to the paper diary traditionally used. 3. Discussion forum for peer support, moderated by the research group. Links to other relevant sites and a section of Frequently Asked Questions also provided. | If women had not accessed the web-based supported in the last fortnight, a reminder is sent out via text message.  A minimum of two independent logons to the system was required for the participant to be considered adhering to the intervention. | n/a | NR | Early pregnancy to 6 months post birth.  Had to log on at least twice to be adherent.  No requirement regarding time spent logged onto system. | None mentioned |  | Wide variation in use, ranging from no individual logins to the equivalent of 15 logins per day.  Of the 78 participants in the ITT analysis of the intervention group, 11 did not fulfil the criteria of two individual logins, leaving usage data from 67 active users to be analysed. |
| **Middleton**  **2021^36^** | Enhanced SMS text-based support & reminder program + standard care | To examine the effectiveness of an enhanced SMS text message–based support and reminder program in improving clinic attendance, metabolic control, engagement in self-management, and psychological health in a young-onset T2DM cohort. | Mobile text messages | Messages were designed to contain a mix of supportive (example given - "Have you noticed sadness or worry getting in the way of your happiness? Talking about it with someone you trust often helps. Your diabetes care team is here for you, so let us know if you are feeling down") and informative (example talks about snacks and diabetes) content. Messages were entirely text-based; emojis and multimedia messaging were not incorporated into the program.  A personalized appointment reminder was sent to participants in the enhanced SMS group in the week before each follow-up appointment. | Mobile text messages managed by an automated system developed and programmed by coauthor AT.  Computer software was run through the University of Sydney Research Electronic Data Capture system. Messages to participants sent through a gateway interface over Australian telephone networks at no cost to individual participants.  All SMS text messages sent were logged by the system; the study log included the date and time that each message was delivered.  There was an option for participants to engage with the study team via the study-specific SMS portal. They could send questions about relating to diabetes and its management directly to the study team at their convenience. The SMS portal was actively monitored by a research assistant, and all text messages received from participants were reviewed and replied to within 1 business day. | n/a | NR | 1^st^ week: intro. message after baseline visit.  1st 2 months: 2 messages a week.  3^rd^ month: 1 message a week  Thereafter: 1 message a month.  Messages were sent at random times during business hours: Monday to Friday, 9AM to 5PM.  Instructions on opting out of the messaging program were provided.  Appointment reminders sent week before follow-up. | Message content individualised based on key baseline characteristics including gender, and smoking status. | n/a | n/a |
| Whiteley  2018^37^ | BattleViro - an iPhone gaming adherence intervention with game related text messages | To examine the preliminary effects of BattleViro - a multilevel gaming intervention for iPhone - on ART adherence, viral load, and relevant knowledge and attitudes among youth living with HIV.  The gaming app aimed to empower youth to improve adherence by increasing information, motivation, and behavioural skills.  The intervention was based on Information Motivation and Behavioural Skills and used an asset model, to promoted self-mastery and social support for adherence.  nb: details about development of intervention given | Mobile phone gaming app | Intervention arm received:   - BattleViro on a smart phone provided by the study, - an electronic pill monitoring device, - twice weekly game related text messages guided by monitoring device data. | In BattleViro, YLWH battle HIV, engage with healthcare providers, and take medication.  Following randomisation, all participants received an electronic pill monitoring device (to establish baseline medication adherence rate). After two weeks, participants returned to receive their study phones, which had a mobile plan for the study period, and their assigned intervention game (BattleViro or non-HIVrelated game).  As participants build skills they move to new, distinctive levels (arterial system, lungs, kidneys, liver, eyes, and brain). Messages from the doctors, nurses and friends encourage and provide clues during difficult twists and turns in the battle. Answering quiz questions from clinician avatars allows players to earn strength and points; wrong answers are corrected and explained.  Participants received adherence-related text messages for the first 8 weeks after receiving their game. Participants received twice weekly game related text messages guided by monitoring device data. Participants with perfect adherence were sent texts of congratulations (e.g., “Great job in Battle. You are winning!”) and others were sent motivational texts (e.g., “Get back in battle! Take your dose!). After the eight-week BattleViro texting period, the adherence text messages stopped, and all participants had 6 additional weeks of use of study phones and their assigned game. | n/a | NR | 14 weeks, as and when | None | n/a | n/a |
