# Supplementary material for: Self‐directed self‐management interventions to prevent or address distress in young people with long‐term physical conditions: A rapid review
Source: Health Expect. 2023 Aug 21;26(6):2164–90. doi: 10.1111/hex.13845 (PMC10632640; doi:10.1111/hex.13845)
Supplement: Supplementary file 3 — Supporting information. [file HEX-26--s001.docx]

**Supplementary file 3: Overall certainty of evidence for intervention characteristics (detailed).** Where reference is in bold italics, the characteristic was not intended to be part of the self-directed intervention i.e. was specific to study only – a sensitivity analysis was conducted to reassess overall certainty of evidence with these references excluded (any changes to individual concerns and overall certainty are indicated).

| **Intervention characteristic** | **References** | **n studies** | **Concerns regarding risk of bias** | **Concerns regarding consistency** | **Concerns regarding precision** | **Concerns regarding applicability** | **OVERALL certainty of evidence** |
| --- | --- | --- | --- | --- | --- | --- | --- |
| **Intervention delivery modes:** | | | | | | | |
| ***Mode used as primary mode only across interventions*** | | | | | | | |
| Web-based (individual) | Ayar 2021, Bell 2021, Chapman 2020, Dilorio 2011, Huang 2014, Joseph 2007, Lam 2020, Linden 2018 | 8 | **Concern:** 5/8 studies have high risk of bias | **Concern:** 3/8 studies show significant benefit | **No concern:** 5/8 studies have *n* ≥ 50 per arm | **Concern:** 1/8 studies has age in range & 1º outcomes of interest | **Low** |
| Mobile device app | Klee 2018, Whiteley 2018 | 2 | **Concern:** 1/2 studies has high risk of bias | **Concern:** 0/2 studies show significant benefit | **Concern:** 0/2 studies has *n* ≥ 50 per arm | **No concern:** 1/2 studies has age in range & 1º outcomes of interest | **Very low** |
| ***Mode used as both primary and secondary modes across interventions*** | | | | | | | |
| Workbook/booklet | Hockenmeyer 2014, Hunt 2020, Lam 2020 | 3 | **Concern:** 2/3 studies have high risk of bias | **Concern:** 1/3 studies show significant benefit | **Concern:** 1/3 studies has *n* ≥ 50 per arm | **Concern:** 0/3 studies has age in range & 1º outcomes of interest | **Very low** |
| Text message | Balatto 2013, Huang 2014, Linden 2018, Middleton 2021, Whiteley 2018 | 5 | **No concern:** 2/5 studies have high risk of bias | **No concern:** 3/5 studies show significant benefit | **Concern:** 1/5 studies has *n* ≥ 50 per arm | **Concern:** 2/5 studies have age in range & 1º outcomes of interest | **Low** |
| ***Mode used as secondary mode only across interventions*** | | | | | | | |
| Web-based (group) | Ayar 2021, Linden 2018 | 2 | **Concern:** 1/2 studies has high risk of bias | **No concern:** 1/2 studies show significant benefit | **No concern:** 1/2 studies has *n* ≥ 50 per arm | **Concern:** 0/2 studies has age in range & 1º outcomes of interest | **Low** |
| Phone call | ***Klee 2018*,** Lam 2020 | 2 | **Concern:** 2/2 studies have high risk of bias | **Concern:** 0/2 studies show significant benefit | **Concern:** 0/2 studies has *n* ≥ 50 per arm | **Concern:** 0/2 studies has age in range & 1º outcomes of interest | **Very low** |
| Email | Klee 2018**, *Lam 2020*** | 2 | **Concern:** 2/2 studies have high risk of bias | **Concern:** 0/2 studies show significant benefit | **Concern:** 0/2 studies has *n* ≥ 50 per arm | **Concern:** 0/2 studies has age in range & 1º outcomes of interest | **Very low** |
| Audio-visual recordings | Chapman 2020, Dilorio 2011, Hockenmeyer 2014 | 3 | **Concern:** 2/3 studies have high risk of bias | **Concern:** 1/3 studies show significant benefit | **No concern:** 2/3 studies have *n* ≥ 50 per arm | **Concern:** 0/3 studies has age in range & 1º outcomes of interest | **Low** |
| Face-to-Face (individual) | Klee 2018 | 1 | **Concern:** 1/1 study has high risk of bias | **Concern:** 0/1 study show significant benefit | **Concern:** 1/1 study has *n <* 50 per arm | **Concern:** 0/1 study has age in range & 1º outcomes of interest | **Very low** |
| ***Number of modes utilised for intervention*** | | | | | | | |
| Single mode | Balatto 2013, Bell 2021, Hunt 2020, Joseph 2007, Middleton 2021 | 5 | **No concern:** 2/5 studies have high risk of bias | **No concern:** 3/5 studies show significant benefit | **No concern:** 3/5 studies have *n* ≥ 50 per arm | **Concern:** 0/5 studies has age in range & 1º outcomes of interest | **Moderate** |
| Combination of modes (≥2 modes) | Ayar 2021, Chapman 2020, Dilorio 2011, Hockenmeyer 2014, Huang 2014, Klee 2018, Lam 2020, Linden 2018, Whiteley 2018 | 9 | **Concern:** 6/9 studies have high risk of bias | **Concern:** 3/9 studies show significant benefit | **Concern:** 3/9 studies have *n* ≥ 50 per arm | **Concern:** 2/9 studies have age in range & 1º outcomes of interest | **Very low** |
| **Additional intervention deliverer (beyond self-administration)** | | | | | | | |
| Health care team/ professionals | Huang 2014, Klee 2018, ***Lam 2020, Middleton 2021*** | 4 | **Concern:** 3/4 studies have high risk of bias | **No concern:** 2/4 studies show significant benefit | **Concern:** 0/4 studies has *n* ≥ 50 per arm | **Concern:** 1/4 studies has age in range & 1º outcomes of interest^1^ | **Low** |
| Automated | Chapman 2020, Huang 2014, Middleton 2021 | 3 | **Concern:** 2/3 studies have high risk of bias | **No concern:** 3/3 study show significant benefit | **Concern:** 1/3 studies has *n* ≥ 50 per arm | **Concern:** 1/3 study has age in range & 1º outcomes of interest | **Low** |
| **Behaviour-change techniques** | | | | | | | |
| CBT components or exercises | Hockenmeyer 2014, Lam 2020 | 2 | **Concern:** 1/2 studies has high risk of bias | **Concern:** 0/2 studies show significant benefit | **Concern:** 0/2 studies has *n* ≥ 50 per arm | **Concern:** 0/2 studies has age in range & 1º outcomes of interest | **Very low** |
| Problem solving | Chapman 2020, Hockenmeyer 2014, Huang 2014, Lam 2020 | 4 | **Concern:** 3/4 studies have high risk of bias | **No concern:** 2/4 studies show significant benefit | **Concern:** 1/4 studies has *n* ≥ 50 per arm | **Concern:** 1/4 studies has age in range & 1º outcomes of interest | **Low** |
| Goal setting | Chapman 2020, Dilorio 2011, Lam 2020 | 3 | **Concern:** 3/3 studies have high risk of bias | **Concern:** 1/3 studies show significant benefit | **No concern:** 2/3 studies have *n* ≥ 50 per arm | **Concern:** 0/3 study has age in range & 1º outcomes of interest | **Low** |
| Action planning | Chapman 2020, Dilorio 2011, Huang 2014, Lam 2020 | 4 | **Concern:** 4/4 studies have high risk of bias | **No concern:** 2/4 studies show significant benefit | **No concern:** 2/4 studies have *n* ≥ 50 per arm | **Concern:** 1/4 studies has age in range & 1º outcomes of interest | **Low** |
| Time management | Lam 2020 | 1 | **Concern:** 1/1 study has high risk of bias | **Concern:** 0/1 study show significant benefit | **Concern:** 1/1 study has *n <* 50 per arm | **Concern:** 0/1 study has age in range & 1º outcomes of interest | **Very low** |
| Brainstorming | Huang 2014 | 1 | **Concern:** 1/1 study has high risk of bias | **No concern:** 1/1 study show significant benefit | **Concern:** 1/1 study has *n <* 50 per arm | **No concern:** 1/1 study has age in range & 1º outcomes of interest | **Low** |
| Pros and cons | Chapman 2020, Dilorio 2011 | 2 | **Concern:** 2/2 studies have high risk of bias | **No concern:** 1/2 studies show significant benefit | **No concern:** 2/2 studies have *n* ≥ 50 per arm | **Concern:** 0/2 studies has age in range & 1º outcomes of interest | **Low** |
| Eliminating avoidance behaviours | Hunt 2020 | 1 | **Concern:** 1/1 study has high risk of bias | **No concern:** 1/1 study show significant benefit | **No concern:** 1/1 study has *n* ≥ 50 per arm | **Concern:** 0/1 study has age in range & 1º outcomes of interest | **Low** |
| Cognitive restructuring (Thought records) | Chapman 2020, Hockenmeyer 2014, Hunt 2020 | 3 | **Concern:** 2/3 studies have high risk of bias | **No concern:** 2/3 studies show significant benefit | **No concern:** 2/3 studies have *n* ≥ 50 per arm | **Concern:** 0/3 study has age in range & 1º outcomes of interest | **Low** |
| Reflect on current behaviours | Dilorio 2011 | 1 | **Concern:** 1/1 studies has high risk of bias | **Concern:** 0/1 studies show significant benefit | **No concern:** 1/1 study has have *n* ≥ 50 per arm | **Concern:** 0/1 study has age in range & 1º outcomes of interest | **Very low** |
| Reflect previous success | Chapman 2020, Lam 2020 | 2 | **Concern:** 2/2 studies have high risk of bias | **No concern:** 1/2 studies show significant benefit | **No concern:** 1/2 studies has *n* ≥ 50 per arm | **Concern:** 0/2 study has age in range & 1º outcomes of interest | **Low** |
| Reminders, prompts & cues (Memory aids) | Ayar 2021, Balatto 2013, Chapman 2020, Dilorio 2011, Huang 2014, Linden 2018, Middleton 2021 | 7 | **Concern:** 5/7 studies have high risk of bias | **No concern:** 5/7 studies show significant benefit | **Concern:** 3/7 studies have *n* ≥ 50 per arm | **Concern:** 1/7 studies has age in range & 1º outcomes of interest | **Low** |
| Feedback | Dilorio 2011, Joseph 2007, ***Lam 2020***, Whiteley 2018 | 4 | **Concern:** 2/4 studies have high risk of bias^1^ | **Concern:** 0/4 studies show significant benefit | **No concern:** 2/4 studies have *n* ≥ 50 per arm | **Concern:** 1/4 studies has age in range & 1º outcomes of interest | **Low** |
| Recording and handling of data including diary | Chapman 2020, Dilorio 2011, Klee 2018, Lam 2020, Linden 2018 | 5 | **Concern:** 4/5 studies have high risk of bias | **Concern:** 1/5 studies show significant benefit | **No concern:** 3/5 studies have *n* ≥ 50 per arm | **Concern:** 0/5 studies has age in range & 1º outcomes of interest | **Low** |
| Behavioural experiments | Hunt 2020 | 1 | **Concern:** 1/1 study has high risk of bias | **No concern:** 1/1 study show significant benefit | **No concern:** 1/1 study has *n* ≥ 50 per arm | **Concern:** 0/1 study has age in range & 1º outcomes of interest | **Low** |
| Credible source | Chapman 2020 | 1 | **Concern:** 1/1 study has high risk of bias | **No concern:** 1/1 study show significant benefit | **No concern:** 1/1 study has *n* ≥ 50 per arm | **Concern:** 0/1 study has age in range & 1º outcomes of interest | **Low** |
| Information about health consequences | Chapman 2020 | 1 | **Concern:** 1/1 study has high risk of bias | **No concern:** 1/1 study show significant benefit | **No concern:** 1/1 study has *n* ≥ 50 per arm | **Concern:** 0/1 study has age in range & 1º outcomes of interest | **Low** |
| Pharmacological support | Chapman 2020 | 1 | **Concern:** 1/1 study has high risk of bias | **No concern:** 1/1 study show significant benefit | **No concern:** 1/1 study has *n* ≥ 50 per arm | **Concern:** 0/1 study has age in range & 1º outcomes of interest | **Low** |
| Direct contact with health care team | Huang 2014 | 1 | **Concern:** 1/1 study has high risk of bias | **No concern:** 1/1 study show significant benefit | **Concern:** 1/1 study has *n <* 50 per arm | **No concern:** 1/1 study has age in range & 1º outcomes of interest | **Low** |
| Signposting to medical care team(s) including allied health | Chapman 2020, Huang 2014, Middleton 2021 | 3 | **Concern:** 2/3 studies have high or unclear risk of bias | **No concern:** 3/3 studies show significant benefit | **Concern:** 1/3 studies has *n* ≥ 50 per arm | **Concern:** 1/3 studies has age in range & 1º outcomes of interest | **Low** |
| Signposting to other social support (family, friends, support groups) | Chapman 2020, Huang 2014, Middleton 2021 | 3 | **Concern:** 2/3 studies have high or unclear risk of bias | **No concern:** 3/3 studies show significant benefit | **Concern:** 1/3 studies has *n* ≥ 50 per arm | **Concern:** 1/3 studies has age in range & 1º outcomes of interest | **Low** |
| Signposting to additional resources, | Chapman 2020, Linden 2018 | 2 | **Concern:** 1/2 studies has high risk of bias | **No concern:** 1/2 studies show significant benefit | **No concern:** 1/2 studies has *n* ≥ 50 per arm | **Concern:** 0/2 studies has age in range & 1º outcomes of interest | **Low** |
| Restructuring of physical environment | Chapman 2020 | 1 | **Concern:** 1/1 study has high risk of bias | **No concern:** 1/1 study show significant benefit | **No concern:** 1/1 study has *n* ≥ 50 per arm | **Concern:** 0/1 study has age in range & 1º outcomes of interest | **Low** |
| Habit formation | Chapman 2020 | 1 | **Concern:** 1/1 study has high risk of bias | **No concern:** 1/1 study show significant benefit | **No concern:** 1/1 study has *n* ≥ 50 per arm | **Concern:** 0/1 study has age in range & 1º outcomes of interest | **Low** |
| Behavioural practice/rehearsal | Chapman 2020 | 1 | **Concern:** 1/1 study has high risk of bias | **No concern:** 1/1 study show significant benefit | **No concern:** 1/1 study has *n* ≥ 50 per arm | **Concern:** 0/1 study has age in range & 1º outcomes of interest | **Low** |
| Demonstration of the behaviour | Chapman 2020 | 1 | **Concern:** 1/1 study has high risk of bias | **No concern:** 1/1 study show significant benefit | **No concern:** 1/1 study has *n* ≥ 50 per arm | **Concern:** 0/1 study has age in range & 1º outcomes of interest | **Low** |
| **Intervention components** | | | | | | | |
| Provision of stress/anxiety specific information | Ayar 2021, Dilorio 2011, Hockenmeyer 2014, Huang 2014, Hunt 2020, Lam 2020, Middleton 2021 | 7 | **Concern:** 5/7 studies have high risk of bias | **No concern:** 4/7 studies show significant benefit | **Concern:** 2/7 studies have *n* ≥ 50 per arm | **Concern:** 1/7 studies has age in range & 1º outcomes of interest | **Low** |
| Materials designed in a simple format | Balatto 2013, Dilorio 2011, Hockenmeyer 2014, Joseph 2007, Klee 2018, Middleton 2021 | 6 | **Concern:** 3/6 studies have high risk of bias | **Concern:** 2/6 studies show significant benefit | **Concern:** 2/6 studies have *n* ≥ 50 per arm | **Concern:** 0/6 studies has age in range & 1º outcomes of interest | **Very low** |
| Tailoring to individual | Chapman 2020, Dilorio 2011, Huang 2014, Joseph 2007, Middleton 2021 | 5 | **Concern:** 3/5 studies have high risk of bias | **No concern:** 3/5 studies show significant benefit | **No concern:** 3/5 studies have *n* ≥ 50 per arm | **Concern:** 1/5 studies has age in range & 1º outcomes of interest | **Low** |
| Disease specific examples and narratives | Bell 2021, Dilorio 2011, Huang 2014, Hunt 2020 | 4 | **Concern:** 3/4 studies have high risk of bias | **No concern:** 2/4 studies show significant benefit | **No concern:** 3/4 studies have *n* ≥ 50 per arm | **Concern:** 1/4 studies has age in range & 1º outcomes of interest | **Low** |
| Blog/Discussion forum | Ayar 2021, Dilorio 2011, Linden 2018 | 3 | **Concern:** 2/3 studies have high risk of bias | **Concern:** 1/3 studies show significant benefit | **No concern:** 2/3 studies have *n* ≥ 50 per arm | **Concern:** 0/3 studies has age in range & 1º outcomes of interest | **Low** |
| Assessment | Dilorio 2011, Lam 2020 | 2 | **Concern:** 2/2 studies have high risk of bias | **Concern:** 0/2 studies show significant benefit | **No concern:** 1/2 studies has *n* ≥ 50 per arm | **Concern:** 0/2 studies has age in range & 1º outcomes of interest | **Very low** |
| Quiz/Poll questions | Ayar 2021, Dilorio 2011, Hunt 2020, Whiteley 2018 | 4 | **Concern:** 3/4 studies have high risk of bias | **No concern:** 2/4 studies show significant benefit | **No concern:** 2/4 studies have *n* ≥ 50 per arm | **Concern:** 1/4 studies has age in range & 1º outcomes of interest | **Low** |
| Writing or drawing exercises | Hockenmeyer 2014, Lam 2020 | 2 | **Concern:** 1/2 studies has high risk of bias | **Concern:** 0/2 studies show significant benefit | **Concern:** 0/2 studies has *n* ≥ 50 per arm | **Concern:** 0/2 studies has age in range & 1º outcomes of interest | **Very low** |
| Exercises (rehabilitation) | Lam 2020 | 1 | **Concern:** 1/1 study has high risk of bias | **Concern:** 0/1 study show significant benefit | **Concern:** 1/1 study has *n <* 50 per arm | **Concern:** 0/1 study has age in range & 1º outcomes of interest | **Very low** |
| Relaxation | Hockenmeyer 2014, Hunt 2020, Lam 2020 | 3 | **Concern:** 2/3 studies have high risk of bias | **Concern:** 1/3 studies show significant benefit | **Concern:** 1/3 studies has *n* ≥ 50 per arm | **Concern:** 0/3 studies has age in range & 1º outcomes of interest | **Very low** |
| Techniques to improve lifestyle | Huang 2014, Hunt 2020 | 2 | **Concern:** 2/2 studies have high risk of bias | **No concern:** 2/2 studies show significant benefit | **No concern:** 1/2 studies has *n* ≥ 50 per arm | **No concern:** 1/2 studies has age in range & 1º outcomes of interest | **Low** |
| Electronic pill monitoring device | Whiteley 2018 | 1 | **No concern:** 1/1 study has low risk of bias | **Concern:** 0/1 study show significant benefit | **Concern:** 1/1 study has *n <* 50 per arm | **No concern:** 1/1 study has age in range & 1º outcomes of interest | **Low** |
|  |  |  |  |  |  |  |  |
|  | **Concern *n* =** | 27 | 48 | 21 | 23 | 46 |  |
|  | **No Concern *n* =** | 24 | 3 | 30 | 28 | 5 |  |
|  |  |  |  |  |  | **High *n* =** | 0 |
|  |  |  |  |  |  | **Moderate *n* =** | 1 |
|  |  |  |  |  |  | **Low *n* =** | 36 |
|  |  |  |  |  |  | **Very low *n* =** | 14 |

^1^ sensitivity analysis *i.e.* excluding study indicated from analysis, led to change from ‘concern’ to ‘no concern’
