# Supplementary material for: Self‐directed self‐management interventions to prevent or address distress in young people with long‐term physical conditions: A rapid review
Source: Health Expect. 2023 Aug 21;26(6):2164–90. doi: 10.1111/hex.13845 (PMC10632640; doi:10.1111/hex.13845)
Supplement: Supplementary file 4 — Supporting information. [file HEX-26--s004.docx]

**Supplementary file 4. Overall certainty of evidence for intervention characteristics without studies with active controls (detailed)**. Italicised characteristics indicate a characteristic affected by removal of studies with active controls. Underlined overall certainty of evidence indicates a change in level.

| **Intervention characteristic** | **References** | **n studies** | **Concerns regarding risk of bias** | **Concerns regarding consistency** | **Concerns regarding precision** | **Concerns regarding applicability** | **OVERALL certainty of evidence** |
| --- | --- | --- | --- | --- | --- | --- | --- |
| **Intervention delivery modes:** | | | | | | | |
| ***Mode used as primary mode only across interventions*** | | | | | | | |
| *Web-based (individual)* | *Ayar 2021, Bell 2021, Chapman 2020, Dilorio 2011, Joseph 2007, Linden 2018* | *6* | ***Concern:*** *3/6 studies have high risk of bias* | ***Concern:*** *2/6 studies show significant benefit* | ***No concern:*** *5/6 studies have n ≥ 50 per arm* | ***Concern:*** *0/6 studies have age in range & 1º outcomes of interest* | ***Low*** |
| Mobile device app | Klee 2018, Whiteley 2018 | 2 | **Concern:** 1/2 studies has high risk of bias | **Concern:** 0/2 studies show significant benefit | **Concern:** 0/2 studies has *n* ≥ 50 per arm | **No concern:** 1/2 studies has age in range & 1º outcomes of interest | **Very low** |
| ***Mode used as both primary and secondary modes across interventions*** | | | | | | | |
| *Workbook/booklet* | *Hockenmeyer 2014* | *1* | ***No concern:*** *1/1 study has low risk of bias* | ***Concern:*** *0/1 study show significant benefit* | ***Concern:*** *1/1 study has n < 50 per arm* | ***Concern:*** *0/1 study have age in range & 1º outcomes of interest* | ***Low^1^*** |
| *Text message* | *Balatto 2013, Linden 2018, Middleton 2021, Whiteley 2018* | *4* | ***No concern:*** *1/4 studies has high risk of bias* | ***No concern:*** *2/4 studies show significant benefit* | ***Concern:*** *1/4 studies has n ≥ 50 per arm* | ***Concern:*** *1/4 studies has age in range & 1º outcomes of interest* | ***Low*** |
| ***Mode used as secondary mode only across interventions*** | | | | | | | |
| Web-based (group) | Ayar 2021, Linden 2018 | 2 | **Concern:** 1/2 studies has high risk of bias | **No concern:** 1/2 studies show significant benefit | **No concern:** 1/2 studies has *n* ≥ 50 per arm | **Concern:** 0/2 studies has age in range & 1º outcomes of interest | **Low** |
| *Phone call* | *Klee 2018* | *1* | ***Concern:*** *1/1 study has high risk of bias* | ***Concern:*** *0/1 studies show significant benefit* | ***Concern:*** *1/1 study has n < 50 per arm* | ***Concern:*** *0/1 study has age in range & 1º outcomes of interest* | ***Very low*** |
| *Email* | *Klee 2018* | *1* | ***Concern:*** *1/1 study has high risk of bias* | ***Concern:*** *0/1 studies show significant benefit* | ***Concern:*** *1/1 study has n < 50 per arm* | ***Concern:*** *0/1 study has age in range & 1º outcomes of interest* | ***Very low*** |
| Audio-visual recordings | Chapman 2020, Dilorio 2011, Hockenmeyer 2014 | 3 | **Concern:** 2/3 studies have high risk of bias | **Concern:** 1/3 studies show significant benefit | **No concern:** 2/3 studies have *n* ≥ 50 per arm | **Concern:** 0/3 studies has age in range & 1º outcomes of interest | **Low** |
| Face-to-Face (individual) | Klee 2018 | 1 | **Concern:** 1/1 study has high risk of bias | **Concern:** 0/1 study show significant benefit | **Concern:** 1/1 study has *n <* 50 per arm | **Concern:** 0/1 study has age in range & 1º outcomes of interest | **Very low** |
| ***Number of modes utilised for intervention*** | | | | | | | |
| *Single mode* | *Balatto 2013, Bell 2021, Joseph 2007, Middleton 2021* | *4* | ***No concern:*** *1/4 studies has high risk of bias* | ***No concern:*** *2/4 studies show significant benefit* | ***No concern:*** *2/4 studies have n ≥ 50 per arm* | ***Concern:*** *0/4 studies has age in range & 1º outcomes of interest* | ***Moderate*** |
| *Combination of modes (≥2 modes)* | *Ayar 2021, Chapman 2020, Dilorio 2011, Hockenmeyer 2014, Klee 2018, Linden 2018, Whiteley 2018* | *7* | ***Concern:*** *4/7 studies have high risk of bias* | ***Concern:*** *2/7 studies show significant benefit* | ***Concern:*** *3/7 studies have n ≥ 50 per arm* | ***Concern:*** *1/7 studies has age in range & 1º outcomes of interest* | ***Very low*** |
| **Additional intervention deliverer (beyond self-administration)** | | | | | | | |
| *Health care team/ professionals* | *Klee 2018, Middleton 2021* | *2* | ***Concern:*** *1/2 studies has high risk of bias* | ***No concern:*** *1/2 studies show significant benefit* | ***Concern:*** *2/2 studies have n < 50 per arm* | ***Concern:*** *0/2 studies has age in range & 1º outcomes of interest (*no concern)* | ***Very low^2^*** |
| *Automated* | *Chapman 2020, Middleton 2021* | *2* | ***Concern:*** *1/2 studies has high risk of bias* | ***No concern:*** *2/2 study show significant benefit* | ***No concern:*** *1/2 studies has n ≥ 50 per arm* | ***No concern:*** *1/2 study has age in range & 1º outcomes of interest* | ***Low*** |
| **Behaviour-change techniques** | | | | | | | |
| *CBT components or exercises* | *Hockenmeyer 2014* | *1* | ***No concern:*** *1/1 study has low risk of bias* | ***Concern:*** *0/1 studies show significant benefit* | ***Concern:*** *1/1 study has n < 50 per arm* | ***Concern:*** *0/1 study has age in range & 1º outcomes of interest* | ***Very low*** |
| *Problem solving* | *Chapman 2020, Hockenmeyer 2014* | *2* | ***Concern:*** *1/2 studies has high risk of bias* | ***No concern:*** *1/2 studies show significant benefit* | ***No concern:*** *1/2 studies has n ≥ 50 per arm* | ***Concern:*** *0/2 studies has age in range & 1º outcomes of interest* | ***Low*** |
| *Goal setting* | *Chapman 2020, Dilorio 2011* | *2* | ***Concern:*** *2/2 studies have high risk of bias* | ***No concern:*** *1/2 studies show significant benefit* | ***No concern:*** *2/2 studies have n ≥ 50 per arm* | ***Concern:*** *0/2 study has age in range & 1º outcomes of interest* | ***Low*** |
| *Action planning* | *Chapman 2020, Dilorio 2011* | *2* | ***Concern:*** *2/2 studies have high risk of bias* | ***No concern:*** *1/2 studies show significant benefit* | ***No concern:*** *2/2 studies have n ≥ 50 per arm* | ***Concern:*** *0/2 study has age in range & 1º outcomes of interest* | ***Low*** |
| *Time management* |  |  |  |  |  |  |  |
| *Brainstorming* |  |  |  |  |  |  |  |
| Pros and cons | Chapman 2020, Dilorio 2011 | 2 | **Concern:** 2/2 study has high risk of bias | **No concern:** 1/2 studies show significant benefit | **No concern:** 2/2 study has *n* ≥ 50 per arm | **Concern:** 0/2 study has age in range & 1º outcomes of interest | **Low** |
| *Eliminating avoidance behaviours* |  |  |  |  |  |  |  |
| *Cognitive restructuring (Thought records)* | *Chapman 2020, Hockenmeyer 2014* | *2* | ***Concern:*** *1/2 studies has high risk of bias* | ***No concern:*** *1/2 studies show significant benefit* | ***No concern:*** *1/2 studies has n ≥ 50 per arm* | ***Concern:*** *0/2 studies has age in range & 1º outcomes of interest* | ***Low*** |
| Reflect on current behaviours | Dilorio 2011 | 1 | **Concern:** 1/1 studies has high risk of bias | **Concern:** 0/1 studies show significant benefit | **No concern:** 1/1 study has have *n* ≥ 50 per arm | **Concern:** 0/1 study has age in range & 1º outcomes of interest | **Very low** |
| *Reflect previous success* | *Chapman 2020* | *1* | ***Concern:*** *1/1 study has high risk of bias* | ***No concern:*** *1/1 studies show significant benefit* | ***No concern:*** *1/1 studies has n ≥ 50 per arm* | ***Concern:*** *0/1 study has age in range & 1º outcomes of interest* | ***Low*** |
| *Reminders, prompts & cues (Memory aids)* | *Ayar 2021, Balatto 2013, Chapman 2020, Dilorio 2011, Linden 2018, Middleton 2021* | *6* | ***Concern:*** *4/6 studies have high risk of bias* | ***No concern:*** *4/6 studies show significant benefit* | ***No concern:*** *3/6 studies have n ≥ 50 per arm* | ***Concern:*** *0/6 studies has age in range & 1º outcomes of interest* | ***Low*** |
| *Feedback* | *Dilorio 2011, Joseph 2007,* *Whiteley 2018* | *3* | ***Concern:*** *1/3 studies have high risk of bias* | ***Concern:*** *0/3 studies show significant benefit* | ***No concern:*** *2/3 studies have n ≥ 50 per arm* | ***Concern:*** *1/3 studies has age in range & 1º outcomes of interest* | ***Low*** |
| *Recording and handling of data including diary* | *Chapman 2020, Dilorio 2011, Klee 2018, Linden 2018* | *4* | ***Concern:*** *3/4 studies have high risk of bias* | ***Concern:*** *1/4 studies show significant benefit* | ***No concern:*** *3/4 studies have n ≥ 50 per arm* | ***Concern:*** *0/4 studies has age in range & 1º outcomes of interest* | ***Low*** |
| *Behavioural experiments* |  |  |  |  |  |  |  |
| Credible source | Chapman 2020 | 1 | **Concern:** 1/1 study has high risk of bias | **No concern:** 1/1 study show significant benefit | **No concern:** 1/1 study has *n* ≥ 50 per arm | **Concern:** 0/1 study has age in range & 1º outcomes of interest | **Low** |
| Information about health consequences | Chapman 2020 | 1 | **Concern:** 1/1 study has high risk of bias | **No concern:** 1/1 study show significant benefit | **No concern:** 1/1 study has *n* ≥ 50 per arm | **Concern:** 0/1 study has age in range & 1º outcomes of interest | **Low** |
| Pharmacological support | Chapman 2020 | 1 | **Concern:** 1/1 study has high risk of bias | **No concern:** 1/1 study show significant benefit | **No concern:** 1/1 study has *n* ≥ 50 per arm | **Concern:** 0/1 study has age in range & 1º outcomes of interest | **Low** |
| *Direct contact with health care team* |  |  |  |  |  |  |  |
| *Signposting to medical care team(s) including allied health* | *Chapman 2020, Middleton 2021* | *2* | ***Concern:*** *1/2 studies has high or unclear risk of bias* | ***No concern:*** *2/2 studies show significant benefit* | ***No concern:*** *1/2 studies has n ≥ 50 per arm* | ***Concern:*** *0/2 studies have age in range & 1º outcomes of interest* | ***Low*** |
| *Signposting to other social support (family, friends, support groups)* | *Chapman 2020, Middleton 2021* | *2* | ***Concern:*** *1/2 studies has high or unclear risk of bias* | ***No concern:*** *2/2 studies show significant benefit* | ***No concern:*** *1/2 studies has n ≥ 50 per arm* | ***Concern:*** *0/2 studies have age in range & 1º outcomes of interest* | ***Low*** |
| Signposting to additional resources, | Chapman 2020, Linden 2018 | 2 | **Concern:** 1/2 studies have high risk of bias | **No concern:** 1/2 studies show significant benefit | **No concern:** 1/2 studies have *n* ≥ 50 per arm | **Concern:** 0/2 studies has age in range & 1º outcomes of interest | **Low** |
| Restructuring of physical environment | Chapman 2020 | 1 | **Concern:** 1/1 study has high risk of bias | **No concern:** 1/1 study show significant benefit | **No concern:** 1/1 study has *n* ≥ 50 per arm | **Concern:** 0/1 study has age in range & 1º outcomes of interest | **Low** |
| Habit formation | Chapman 2020 | 1 | **Concern:** 1/1 study has high risk of bias | **No concern:** 1/1 study show significant benefit | **No concern:** 1/1 study has *n* ≥ 50 per arm | **Concern:** 0/1 study has age in range & 1º outcomes of interest | **Low** |
| Behavioural practice/rehearsal | Chapman 2020 | 1 | **Concern:** 1/1 study has high risk of bias | **No concern:** 1/1 study show significant benefit | **No concern:** 1/1 study has *n* ≥ 50 per arm | **Concern:** 0/1 study has age in range & 1º outcomes of interest | **Low** |
| Demonstration of the behaviour | Chapman 2020 | 1 | **Concern:** 1/1 study has high risk of bias | **No concern:** 1/1 study show significant benefit | **No concern:** 1/1 study has *n* ≥ 50 per arm | **Concern:** 0/1 study has age in range & 1º outcomes of interest | **Low** |
| **Intervention components** | | | | | | | |
| *Provision of stress/anxiety specific information* | *Ayar 2021, Dilorio 2011, Hockenmeyer 2014, Middleton 2021* | *4* | ***Concern:*** *2/4 studies have high risk of bias* | ***No concern:*** *2/4 studies show significant benefit* | ***Concern:*** *1/4 studies have n ≥ 50 per arm* | ***Concern:*** *0/4 studies has age in range & 1º outcomes of interest* | ***Low*** |
| Materials designed in a simple format | Balatto 2013, Dilorio 2011, Hockenmeyer 2014, Joseph 2007, Klee 2018, Middleton 2021 | 6 | **Concern:** 3/6 studies have high risk of bias | **Concern:** 2/6 studies show significant benefit | **Concern:** 2/6 studies have *n* ≥ 50 per arm | **Concern:** 0/6 studies have age in range & 1º outcomes of interest | **Very low** |
| *Tailoring to individual* | *Chapman 2020, Dilorio 2011, Joseph 2007, Middleton 2021* | *4* | ***Concern:*** *2/4 studies have high risk of bias* | ***No concern:*** *2/4 studies show significant benefit* | ***No concern:*** *3/4 studies have n ≥ 50 per arm* | ***Concern:*** *0/4 studies has age in range & 1º outcomes of interest* | ***Low*** |
| *Disease specific examples and narratives* | *Bell 2021, Dilorio 2011* | *2* | ***Concern:*** *1/2 studies has high risk of bias* | ***Concern:*** *0/2 studies show significant benefit* | ***No concern:*** *2/2 studies have n ≥ 50 per arm* | ***Concern:*** *1/2 studies has age in range & 1º outcomes of interest* | ***Very low^2^*** |
| Blog/Discussion forum | Ayar 2021, Dilorio 2011, Linden 2018 | 3 | **Concern:** 2/3 studies have high risk of bias | **Concern:** 1/3 studies show significant benefit | **No concern:** 2/3 studies have *n* ≥ 50 per arm | **Concern:** 0/3 studies has age in range & 1º outcomes of interest | **Low** |
| *Assessment* | *Dilorio 2011* | *1* | ***Concern:*** *1/1 studies has high risk of bias* | ***Concern:*** *0/1 studies show significant benefit* | ***No concern:*** *1/1 study has have n ≥ 50 per arm* | ***Concern:*** *0/1 study has age in range & 1º outcomes of interest* | ***Very low*** |
| *Quiz/Poll questions* | *Ayar 2021, Dilorio 2011, Whiteley 2018* | *3* | ***Concern:*** *2/3 studies have high risk of bias* | ***Concern:*** *1/3 studies show significant benefit* | ***Concern:*** *1/3 studies has n ≥ 50 per arm* | ***Concern:*** *1/3 studies has age in range & 1º outcomes of interest* | ***Very low^2^*** |
| *Writing or drawing exercises* | *Hockenmeyer 2014* | *1* | ***No concern:*** *1/1 study has low risk of bias* | ***Concern:*** *0/1 studies show significant benefit* | ***Concern:*** *1/1 study has n < 50 per arm* | ***Concern:*** *0/1 study has age in range & 1º outcomes of interest* | ***Very low*** |
| *Exercises (rehabilitation)* |  |  |  |  |  |  |  |
| *Relaxation* | *Hockenmeyer 2014* | *1* | ***No concern:*** *1/1 study has low risk of bias* | ***Concern:*** *0/1 studies show significant benefit* | ***Concern:*** *1/1 study has n < 50 per arm* | ***Concern:*** *0/1 study has age in range & 1º outcomes of interest* | ***Very low*** |
| *Techniques to improve lifestyle* |  |  |  |  |  |  |  |
| Electronic pill monitoring device | Whiteley 2018 | 1 | **No concern:** 1/1 study has low risk of bias | **Concern:** 0/1 study show significant benefit | **Concern:** 1/1 study has *n <* 50 per arm | **No concern:** 1/1 study has age in range & 1º outcomes of interest | **Low** |
|  |  |  |  |  |  |  |  |
|  | **Concern *n* =** | 30 | 37 | 20 | 15 | 41 |  |
|  | **No Concern *n* =** | 14 | 7 | 24 | 29 | 3 |  |
|  |  |  |  |  |  | **High *n* =** | 0 |
|  |  |  |  |  |  | **Moderate *n* =** | 1 |
|  |  |  |  |  |  | **Low *n* =** | 29 |
|  |  |  |  |  |  | **Very low *n* =** | 14 |

^1^upgraded from very low to low certainty of evidence; ^2^downgraded from low to very low level of certainty
